# Mechanisms of Water-Lock Removal by a Nanoemulsion/Mud Acid Composite System in Low-Permeability Sandstone Reservoirs: Insights from Pore-Scale Characterization in the MB Block

**DOI:** 10.1021/acsomega.6c07390

**Published:** 2026-09-29

**Authors:** Guohui Qu, Jingxuan Wu, Michael Zhengmeng Hou, Yikun Liu, Louis Osei Twum, Ning Liu

**Affiliations:** † Ministry of Education, Key Laboratory of Enhanced Oil Recovery (Northeast Petroleum University), Daqing, Heilongjiang 163318, China; ‡ 26534 Clausthal University of Technology, Clausthal-Zellerfeld 38678, Germany; § 4365 University of Louisiana at Lafayette, Lafayette, Louisiana 70504, United States

## Abstract

Water-lock damage in low-permeability sandstone reservoirs is primarily caused by small pore-throat structures, strong capillary forces, and hydrophilic rock surfaces. Conventional treatment methods mainly target either fluid-interface modification or pore blockage problems separately. In this study, a nanoemulsion/mud acid composite system was developed to achieve a combined effect of fluid interfaces and localized mineral dissolution. Interfacial tension (IFT) measurements, contact angle analysis, constant-rate mercury intrusion (CRMI), nuclear magnetic resonance (NMR), and high-temperature high-pressure core flooding experiments were conducted to investigate the water-lock removal performance and underlying mechanisms of the composite system. The results demonstrate that the optimized 0.20 wt % nanoemulsion/mud acid system reduced the oil–water interfacial tension to 0.486 × 10^–3^ (mN·m^–1^) and increased the contact angle to 116.4°. The combined CRMI–NMR characterization revealed that fluid mobility in low-permeability sandstone can be classified into bound, transition, and movable zones, rather than being described by a single flow threshold. Increasing the production pressure differential intensified deep water-phase invasion and retention, weakened the production enhancement effect caused by increased driving force, and further aggravated water-lock damage. Mineralogical analysis, NMR measurements, and permeability-recovery experiments collectively indicate that water-lock removal is associated with coupled interfacial regulation and localized pore–throat modification. These findings provide a pore-scale theoretical basis for optimizing treatment-agent concentration and production pressure differential, while further field-scale validation remains necessary.

## Graphical abstract

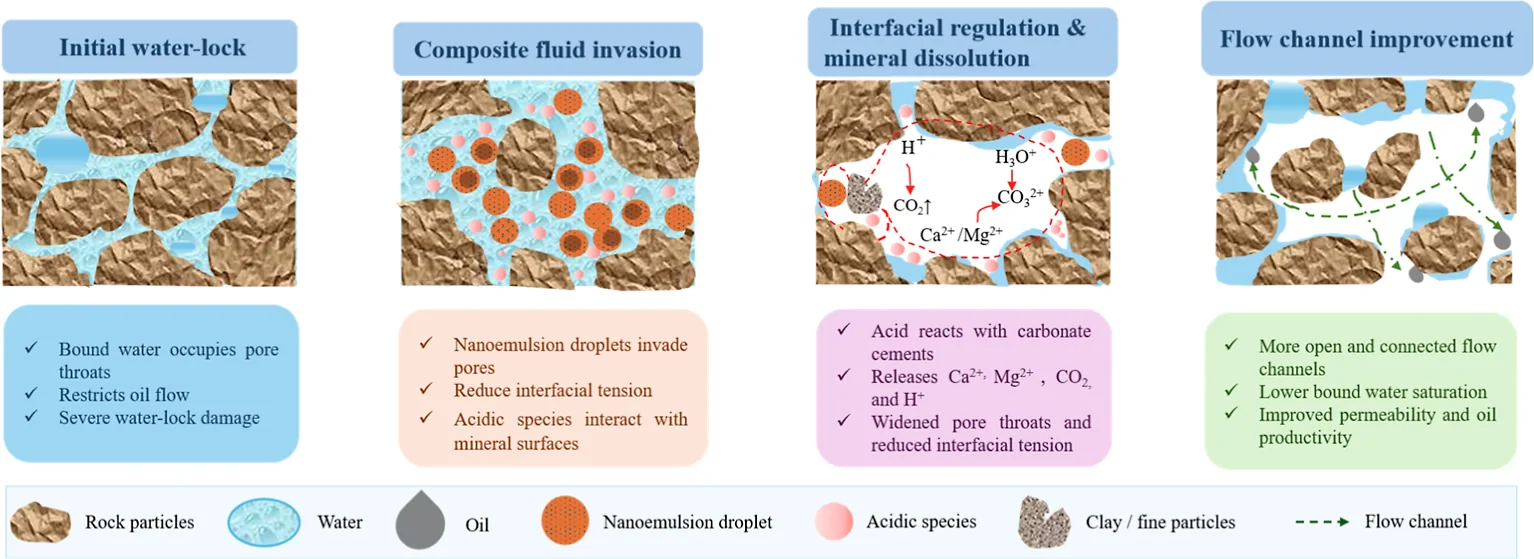

## 1 Introduction

Water-lock damage is a major cause of permeability impairment and productivity decline in low-permeability sandstone reservoirs. Fine pore throats, strong water affinity, and high capillary resistance promote aqueous-phase retention and restrict hydrocarbon flow. The severity of this damage depends not only on the intrinsic pore-throat structure but also on interfacial properties and the volume and depth of water invasion during production or stimulation.^1–3^

Existing water-lock mitigation approaches can be classified according to the principal problem that they address. Interfacial-regulation methods employ surfactants, nanoemulsions, or nanofluids to reduce interfacial tension and modify rock-water affinity, thereby weakening capillary retention and facilitating the displacement or flowback of retained water.^4–6^ Multiscale investigations further indicate that the effectiveness of such treatments depends not only on their interfacial activity but also on effective pore volume, throat accessibility, pore–throat connectivity, mineral filling or cementation, and pore-network heterogeneity.^7–9^ Consequently, formulations that only modify interfacial properties may have limited effectiveness where fluid transport is additionally restricted by mineral or particulate blockage.

Acid-induced pore-throat modification provides another route for restoring sandstone flow capacity. Mud acid generally consists of hydrochloric acid and hydrofluoric acid: hydrochloric acid dissolves carbonate minerals and maintains an acidic reaction environment, whereas hydrofluoric acid reacts primarily with feldspars, clay minerals, and other silicate components. Emulsified acids, associative-polymer acids, and surfactant-retarded HF/HCl formulations have therefore been developed to reduce premature acid consumption, extend the effective treatment distance, and improve pore-throat connectivity.^10–13^ However, under elevated-temperature conditions, accelerated acid–rock reactions may cause premature acid spending and limited penetration, while increased corrosion and secondary or tertiary precipitation may induce additional formation damage.^14–16^ These limitations indicate that acidizing performance should be evaluated together with interfacial behavior and pore-scale fluid redistribution rather than solely through the final permeability increase.

Despite these advances, several issues remain insufficiently resolved. First, nanoemulsion treatments and sandstone acidizing have commonly been evaluated as separate strategies: the former primarily targets capillary water retention, whereas the latter mainly addresses mineral or solid blockage. Although surfactant-acid formulations have been investigated for acid retardation, injectivity improvement, and permeability enhancement, their contribution to water-lock mitigation has rarely been quantified simultaneously from interfacial, pore-structural, and fluid-mobility perspectives. Second, existing studies generally characterize fluid mobility using bulk recovery indices or a single pore-size cutoff, whereas heterogeneous low-permeability sandstones may contain a gradual transition from bound to movable fluids. Third, the production pressure differential has a dual influence: increasing the driving force can promote fluid production, but it can also increase the volume and depth of aqueous-phase invasion and thereby aggravate water retention. A multiscale framework linking interfacial properties, mineral alteration, pore–throat accessibility, fluid-mobility zoning, and pressure-dependent permeability recovery is therefore still required.

Unlike previous surfactant-acid studies focused primarily on acid retardation, injectivity, and macroscopic permeability enhancement, pore-scale investigations linking interfacial performance and mineral alteration to constant-rate mercury intrusion (CRMI) and nuclear magnetic resonance (NMR)-derived fluid-mobility zones and pressure-dependent water-lock damage remain limited. To fill this gap, this work develops a nanoemulsion/mud-acid composite system and constructs a pore-scale evaluation framework for water-lock remediation using low-permeability sandstone cores from the Sangonghe Formation in MB Block of the Karamay Oilfield. Integrating interfacial testing, CRMI/NMR joint characterization, mineralogical analysis and core flooding experiments, the study first optimizes the composite concentration via interfacial tension and contact angle measurements, then partitions fluid mobility zones and identifies the movable fluid threshold through CRMI/NMR, and finally quantifies water-lock damage and permeability recovery under varying production pressure differentials. This work evaluates how interfacial regulation and mineral alteration jointly contribute to water-lock remediation by linking interfacial-tension reduction, wettability response, localized mineral modification, fluid-mobility redistribution, and permeability recovery. The proposed mechanism of water lock removal by the nanoemulsion/mud-acid composite system is schematically illustrated in Figure 1. The core contribution lies in the proposed pore scale evaluation framework rather than in claiming a universally superior surfactant acid formulation.

**Figure 1:**
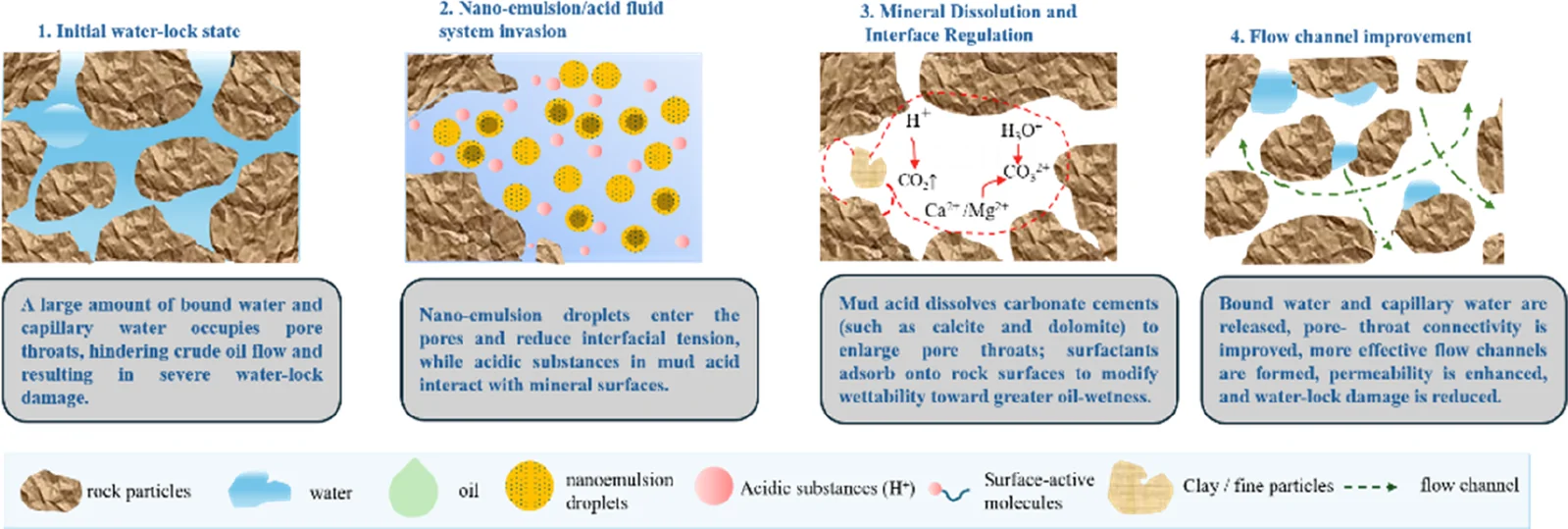
Mechanism diagram of nanoemulsion/acid fluid for water-blocking removal.

## 2 Materials and Methods

### 2.1 Experimental Preparation

The chemical reagents used in the experiments are listed in Table 1.

**Table 1:** Specifications and Manufacturers of Experimental Reagents

no	chemical name	specification	manufacturer
1	perfluorodecyltrimethylsilane (PDTS1)	97%	Shanghai Aladdin Biochemical Technology Co., Ltd.
2	penetrant JFC	industrial grade	Shanghai Tiantan Auxiliaries Co., Ltd.
3	D-limonene	industrial grade	Hunan Honghua Jinjia Spice Co., Ltd.
4	sodium chloride (NaCl)	analytical grade	Shanghai Yuanye Bio-Technology Co., Ltd.
5	*n*-pentanol	analytical grade	Dekens Chemical (Shandong) Co., Ltd.
6	acid solution	3% HF + 10% HCl	laboratory-prepared
7	deionized water	-	laboratory-prepared
8	simulated formation water	-	simulated formation water, laboratory-prepared according to the ionic composition of formation water from MB Block.
9	heavy water(D_2_O)	-	J&K Scientific Ltd., Beijing

Experimental oil: The experimental oil was simulated oil prepared by mixing dehydrated and degassed crude oil produced from the low-permeability reservoir in MB Block of the Xinjiang Oilfield with kerosene. Its viscosity was 0.74 mPa·s at 105 °C. The porosity and permeability of the four core samples were measured by helium adsorption experiments and the pulse-decay method, respectively, as shown in Table 2.

**Table 2:** Physical Property Parameters of Low-Permeability Sandstone Core Samples in the Study Area

core sample	coring horizon	diameter (mm)	length (mm)	depth interval (m)	reservoir temperature (°C)	permeability (10^–3^ μm^2^)	porosity (%)
26–1	M5	24.71	49.42	4052.60–4055.00	104.50	0.44	13.64
17–2	M4	24.71	50.00	4230.80–4230.90	103.20	0.83	14.11
9–2	M6	24.71	49.62	3993.8–3994.0	101.90	1.568	15.27
11–1	M8	24.71	49.22	3998–4000	102.20	3.5	18.63

#### 2.1.1 Preparation of the Nanoemulsion/Acid System

First, 3 wt % perfluorodecyltrimethylsilane (PDTS1), 3 wt % nonionic penetrant JFC, 3 wt % d-limonene, and 6 wt % *n*-pentanol were added to deionized water at a mass ratio of 1:1:1:2. The component ratio was selected based on preliminary formulation screening and was kept constant in the present study because the subsequent experiments focused on optimizing the total working concentration rather than reoptimizing the internal component ratio. The mixture was magnetically stirred at 5000 rpm for 120 min at 60 °C to ensure sufficient dispersion and form the nanoemulsion stock solution. Subsequently, mud acid was added according to the designed proportion, and the mixture was continuously stirred for another 60 min to obtain the nanoemulsion/acid composite system. The initial nanoemulsion/acid composite system was then diluted with simulated formation water to prepare systems with different mass fractions of 0.05, 0.10, 0.15, 0.20, 0.25, and 0.30 wt %.

### 2.2 Experimental Methods

#### 2.2.1 Performance Testing of the Nanoemulsion/Acid Composite System

##### 2.2.1.1 Temperature and Salinity Resistance Test

The temperature and salinity resistance of the prepared nanoemulsion/acid system was evaluated using a laser particle size analyzer. Simulated formation water with a salinity of (1.5 × 10^5^) mg/L was prepared using sodium chloride. Then, 0.1 wt % nanoemulsion/acid solution was added to the simulated formation water, and the solution was placed in an oven at 150 °C. The particle size of the solution was measured after aging for 0, 5, 10, 15, 20, 25, 30, 35, and 40 days. A concentration of 0.10 wt % was selected for the temperature- and salinity-resistance test because it provided sufficient scattering intensity for reliable particle-size measurements while minimizing multiple-scattering errors associated with higher droplet concentrations.

##### 2.2.1.2 Interfacial Tension Measurement

In this study, the interfacial tension between nanoemulsion/acid systems with different concentrations and simulated oil was measured using an SVT-20N video spinning drop tensiometer, with a measurement range of (1 × 10^–6^) to (2 × 10^3^) (mN·m^–1^) and a temperature control range of −10 to 130 °C. The tests were conducted at 105 °C and a rotational speed of 5000 rpm. A high-resolution imaging system was used to capture the morphology of the oil droplets in real time, and the interfacial tension was continuously monitored for 120 min.

##### 2.2.1.3 Contact Angle Measurement

Oil-saturated sandstone slices were immersed in nanoemulsion/mud-acid systems with different concentrations and aged at 105 °C for 72 h. After removal of surface liquid and drying, simulated formation-water droplets were placed on the sample surfaces. Apparent static water contact angles were measured at room temperature using baseline circle fitting. The measurements were used for relative comparison of the wettability responses among the tested treatments.

The contact angle was determined directly from the sessile–drop profile using the baseline circle-fitting method and is reported as an apparent static water contact angle. It was not calculated using either the Young or Wenzel equation. The Young equation applies to an ideal smooth and chemically homogeneous surface, whereas application of the Wenzel model requires a known surface-roughness ratio and complete liquid penetration into the surface asperities. Because surface roughness was not quantitatively measured in this study, no Wenzel correction was applied. The measured contact angles were therefore used to compare the relative wettability responses of different treatments under identical measurement conditions.

#### 2.2.2 Pore Throat Distribution Characteristics and the Lower Limit of Movable Fluids

The technical workflow of the combined CRMI/NMR method is shown in Figure 2.

**Figure 2:**
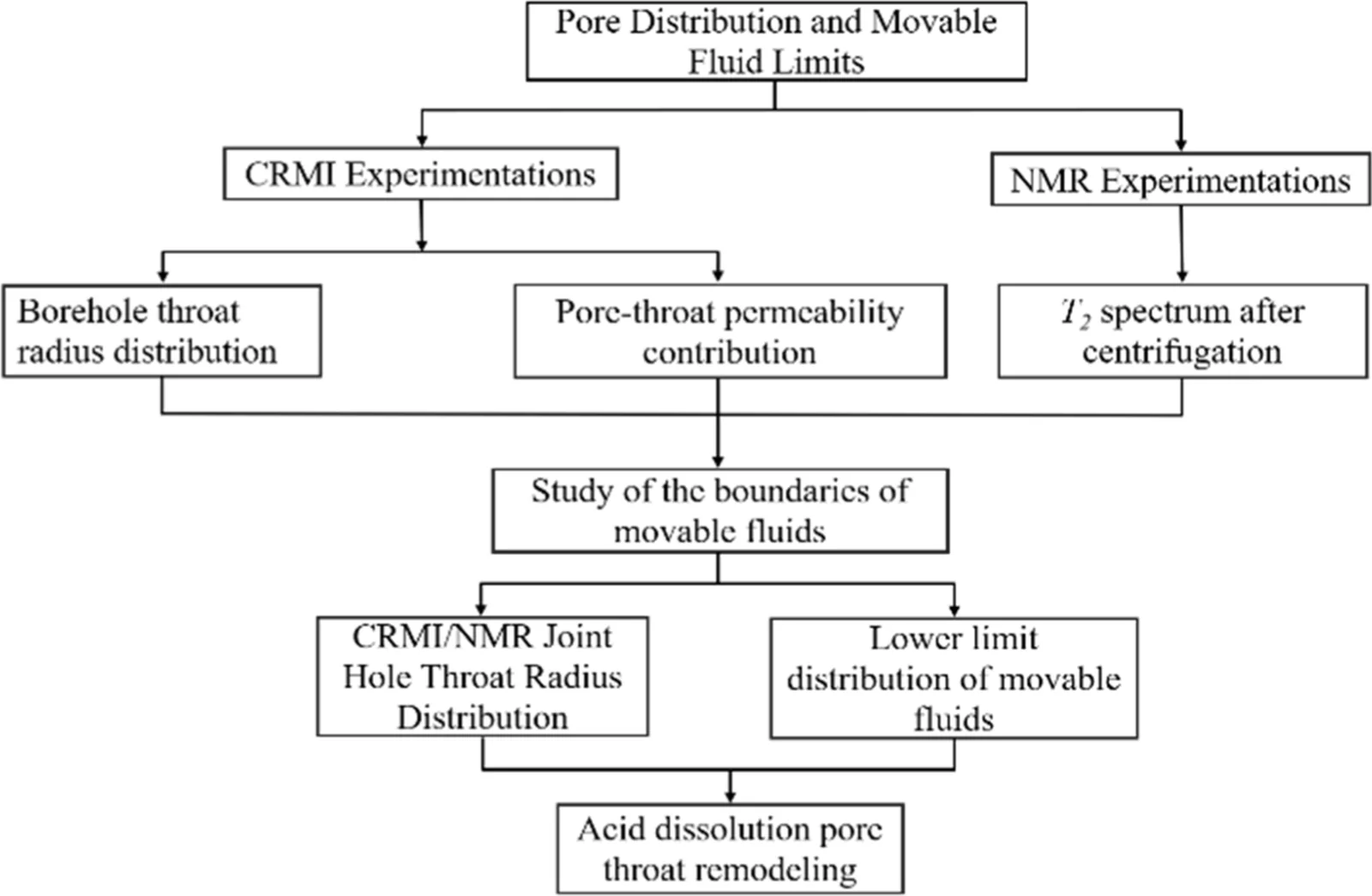
Technical roadmap for CRMI/NMR combined method.

CRMI and NMR measurements were combined to characterize the throat-related intrusion response and pore-body-related fluid distribution of the representative core samples. The experimental procedures and data-conversion method are described below.

##### 2.2.2.1 Constant-Rate Mercury Intrusion Experiment

After oil removal and drying, representative core plugs were analyzed using a constant-rate mercury-intrusion porosimeter. Mercury was injected at a constant rate of 0.0001 mL/min until the preset maximum pressure was reached, then the pressure was reduced under control. The pore-body radius, throat radius, mercury-saturation, and permeability-contribution distributions were obtained using the instrument’s pore–throat separation model. The mercury–air interfacial tension and contact angle were set to 480 (mN·m^–1^) and 140°, respectively.

##### 2.2.2.2 Nuclear Magnetic Resonance Experiment

Water-saturated cores were scanned to obtain the initial *T*_2_ spectra and were subsequently centrifuged at 10,000 rpm for 20 min to establish the retained-water state. A second *T*_2_ measurement was then performed under the same acquisition parameters. The difference between the saturated and postcentrifugation spectra was used to characterize the movable-fluid response.

According to the surface-relaxation model, the transverse relaxation time *T*_2_ is related to the characteristic pore size as follows^17^

1 $$T_{2}=\frac{1}{\rho }\frac{V}{S}=\frac{1}{\rho }\frac{r}{c}$$

where ρ is the surface relaxivity (μm/ms), *V*/*S* is the volume-to-surface area ratio of pores, *c* is the pore shape factor, and *r* is the pore radius (μm). The detailed calibration of surface relaxivity and the geometric assumptions used for the CRMI–NMR conversion are discussed in Section 3.2.3.

#### 2.2.3 Quantitative Evaluation of Water Locking Damage

After saturating the core with heavy water, oil is injected to establish an oil-saturated state with bound heavy water. The core is aged for 24 h, and the *T*_2_ spectrum is acquired under bound-water conditions. During the experiment, the inlet pressure and confining pressure are maintained at 35 and 38 MPa, respectively. The outlet backpressure is reduced stepwise from 30 to 5 MPa, corresponding to production pressure differences of 5 MPa, 10 MPa, 15 MPa, 20 MPa, 25 and 30 MPa. At each pressure-difference stage, displacement continues until the outlet fluid production and pressure become stable. The oil production, water production and effective permeability are then recorded, and the NMR *T*_2_ test is performed; the experimental flow is shown in Figure 3. The degree of reservoir damage caused by water locking under different pressure differentials was calculated using eq 2, and to facilitate comparison among cores with different initial permeabilities, the following dimensionless water-locking (*I*_k_) damage coefficient was defined in this study

2 $$I_{k}=\frac{\left(K_{D}-K_{f}\right)}{K_{D}}$$

where *I*_k_ is the water locking damage coefficient; *K*_D_ is the permeability of the core after oil washing and drying, 10^–3^ μm^2^; and *K*_f_ is the permeability of the core after water locking damage, 10^–3^ μm^2^.

**Figure 3:**
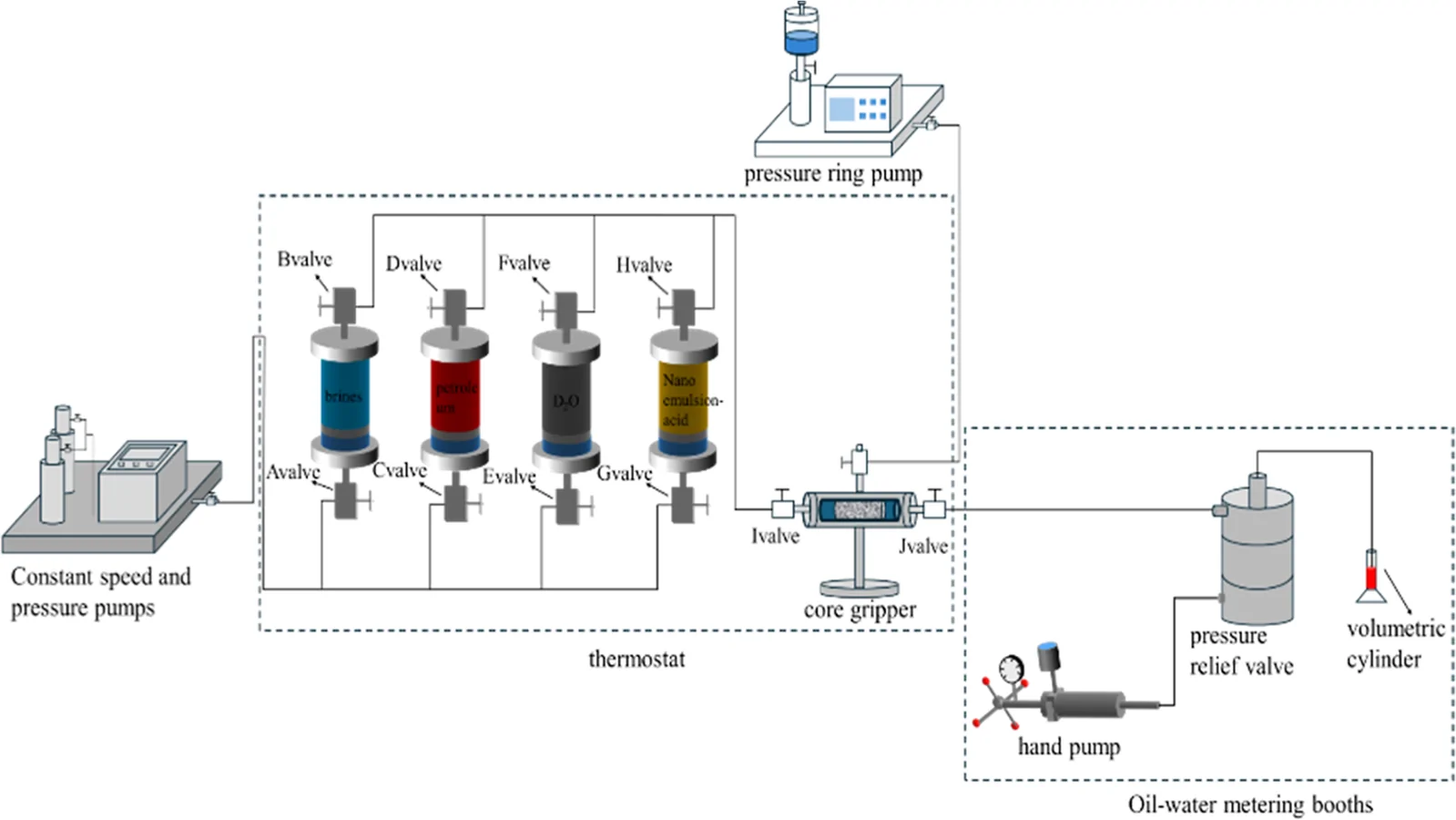
Schematic diagram of the experimental apparatus.

#### 2.2.4 Quantitative Evaluation of Water Locking Recovery

The nanoemulsion/acid system was prepared using heavy water as the aqueous phase and used as the displacement agent. The experimental procedures were the same as steps ①–④ in Section 2.2.3. To facilitate the comparison of core permeability after water lock removal, this study defines the following dimensionless water lock recovery coefficient (*I*_a_): eq 3

3 $$I_{a}=\frac{\left(K_{m}-K_{f}\right)}{K_{m}}$$

where *I*_a_ is the water-locking recovery coefficient; *K*_m_ is the restored permeability of the core after water-lock removal, 10^–3^ μm^2^; and *K*_f_ is the permeability of the core after water-locking damage, 10^–3^ μm^2^.

## 3 Results and Discussion

### 3.1 Performance Evaluation of the Nanoemulsion/Acid System

#### 3.1.1 Stability Evaluation of the Nanoemulsion/Acid System

As shown in Figure 4, the particle size of the nanoemulsion system increased slowly with increasing standing time. The initial particle size was approximately 35.7 nm and increased to about 39.8 nm after 40 days, indicating only a slight increase in particle size. This suggests that only minor aggregation or droplet growth occurred during long-term aging. Throughout the entire test period, the particle size remained below 50 nm, still within the nanoscale range, indicating limited particle-size growth and no apparent loss of nanoscale dispersion during the tested aging period.

**Figure 4:**
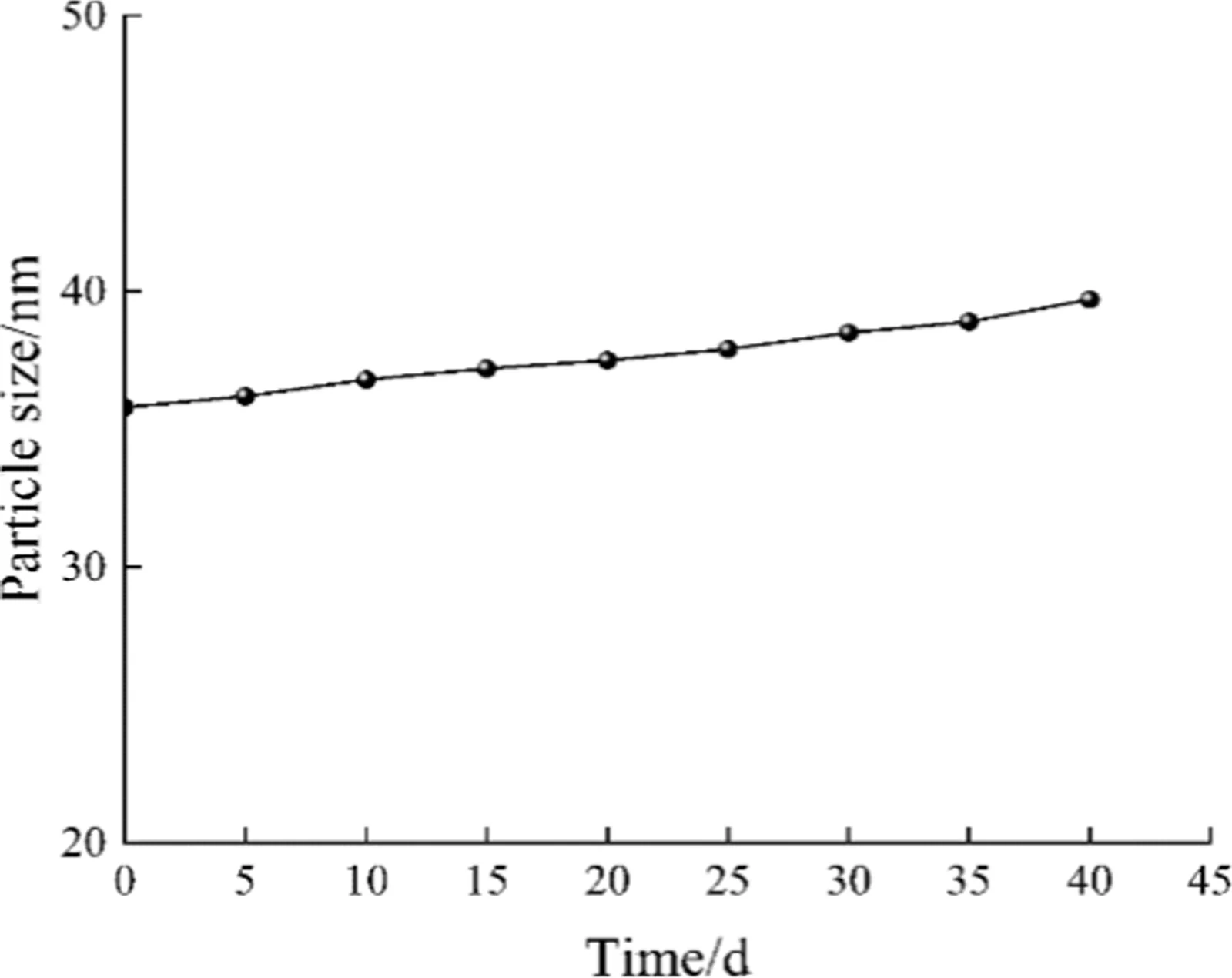
Evolution law of average particle size of nanoemulsion/mud acid system with mass fraction of 0.10 wt % aged at 150 °C in simulated formation water with salinity of 1.5 × 10^5^ mg/L.

The slow increase in particle size may be related to Brownian motion induced collisions between droplets, rearrangement of the interfacial film, and limited coalescence of small droplets.^19,20^ No visible phase separation or severe aggregation was observed during the aging period, and the mean droplet size remained within the nanoscale range, indicating that the composite formulation maintained acceptable kinetic stability over the investigated period. Such stability is a prerequisite for maintaining droplet dispersion and interfacial activity during injection. Such stability helps preserve the dispersed state and interfacial activity of the droplets during injection. Combined with the measured droplet size, interfacial tension, contact angle, and core-flooding results, the nanoscale dispersion may facilitate transport through accessible pore throats and contribute to interfacial-tension reduction, wettability alteration, and water-lock mitigation.^4^ Particle-size evolution is an important indicator of the kinetic stability of nanoemulsion systems. In the present study, the mean droplet size increased only slightly from 35.7 to 39.8 nm during aging, corresponding to a relative increase of approximately 11.5%, while no visible phase separation or severe aggregation was observed. These results indicate that the composite system maintained acceptable colloidal stability within the investigated period.^21^ However, particle-size stability alone cannot confirm the preservation of interfacial-tension reduction, wettability alteration, or core-scale water-lock-removal performance, which should be evaluated independently through aged interfacial-tension, contact-angle, and core-flooding measurements.

Recently reported nanoemulsion systems for sandstone-reservoir applications span a relatively broad droplet-size range, from several tens of nanometers to above 100 nm.^4^ After 40 days of aging, the particle size of the system in this study remains between 35.7 and 39.8 nm, indicating that the particle growth of the system is limited under high-temperature and high-salinity conditions. By contrast, the KH550-modified nanosilica incorporated into a recently reported fluorosurfactant composite system exhibited an average particle size of approximately 40 nm.^22^ Because nanoparticles and nanoemulsion droplets represent different dispersed phases, their particle sizes cannot be directly compared on a strictly quantitative basis. In this work, the initial particle size of the nanoemulsion/mud acid composite system was 35.7 nm. After 40 days of aging at 150 °C and 1.5 × 10^5^ mg/L salinity, the particle size increased to 39.8 nm, with a relative growth rate of approximately 11.5%, and the particle size always remained below 50 nm, confirming that the system has favorable dispersion stability and can satisfy the migration requirement in fine pore throats of low-permeability reservoirs.

#### 3.1.2 Interfacial Tension Measurement

As shown in Figure 5, the interfacial tension decreased with increasing mass fraction of the nanoemulsion/acid system. The dynamic interfacial tension curves of the 0.20 and 0.30 wt % systems exhibit a slope change at approximately 40 min. This change may reflect the transition of interfacially active components from the early stage rapid diffusion and adsorption to the stage of gradually increased interfacial coverage and slow rearrangement of the interfacial film. Within 0–50 min, the interfacial tension decreased rapidly with time. After 50 min, adsorption equilibrium was gradually reached, and the interfacial tension tended to stabilize. The nanoemulsion/acid system exhibited the lowest interfacial tension at a mass fraction of 0.20 wt %, and the interfacial tension was lower than 0.001 (mN·m^–1^), reaching the ultralow interfacial tension range. At this stage, the adsorption amount of surface-active molecules at the oil/water interface increased with concentration, resulting in a rapid decrease in interfacial tension.

**Figure 5:**
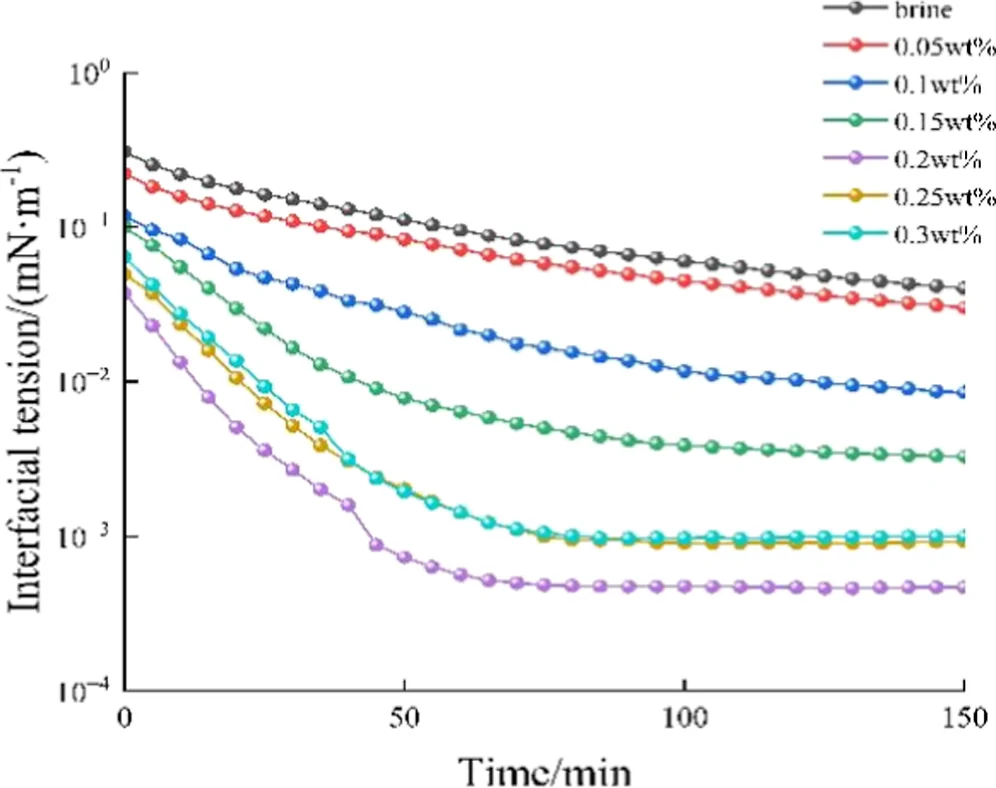
Dynamic oil–water interfacial tension of nanoemulsion/mud-acid systems with different concentrations measured at 105 °C and 5000 rpm for 120 min.

When the concentration was lower than 0.20 wt %, the adsorption amount of active components at the oil–water interface increased with increasing concentration, leading to a substantial reduction in interfacial tension. When the concentration approached 0.20 wt %, interfacial adsorption tended to become saturated. Further increasing the concentration mainly promoted micelle formation, contributing only slightly to additional interfacial tension reduction. Meanwhile, a high-concentration system may increase viscosity and the risk of retention in pore throats.

For the 0.20 wt % nanoemulsion/acid system, the interfacial tension reached 0.486 × 10^–3^ (mN·m^–1^) at 75 min, and no obvious further decrease was observed after 75 min. As a cosurfactant, n-Pentanol features favorable acid resistance and a small molecular size. It can insert into the oil–water interfacial film to adjust the hydrophilic–lipophilic balance of the system, reduce the rigidity of the interfacial adsorption layer, and accelerate the rapid migration of active molecules from micelles to the interface, thereby achieving a swift reduction in oil–water interfacial tension. Unlike conventional long-chain cosurfactants, n-pentanol does not increase the particle size of emulsion droplets. Moreover, it maintains stable chemical properties under high-concentration mud acid and high-temperature conditions, satisfying the application requirements for tiny pore throats in low-permeability reservoirs and acidizing stimulation operations. In addition, different from conventional alkane oil phases that only exert a simple dilution effect, d-limonene can penetrate into crude oil to weaken the association of heavy components such as resins and asphaltenes, reduce the oil–water interfacial energy, and synergistically accelerate the reduction of interfacial tension. Meanwhile, d-limonene maintains stable chemical properties in high-concentration mud acid and high-temperature environments, which matches the application requirements of the nanoemulsion/acid composite system. The minimum oil - water interfacial tension obtained in this study was 0.486 × 10^–3^ (mN·m^–1^). Under the respective experimental conditions, this value is lower than those reported for selected recently developed nanoemulsion and green nanofluid formulations for low-permeability sandstone reservoirs.^22,23^ Nevertheless, because interfacial tension depends strongly on crude-oil composition, temperature, salinity, formulation concentration, and measurement protocol, this comparison should be regarded as indicative rather than as evidence of universal performance superiority. For instance, one type of wetting regulator can reduce the oil–water interfacial tension to approximately 0.11 (mN·m^–1^), while green nanoemulsion flooding systems enhance crude oil recovery through the combined effect of interfacial activity and emulsion stability. The interfacial tension value obtained in this study is markedly lower, which may stem from the combined effect of fluorine-containing surfactant components, cosurfactants, d-limonene, and the mud acid environment.

Nevertheless, interfacial tension values from different studies cannot be directly adopted to evaluate the performance of formulations, as discrepancies exist in crude oil components, aqueous phase salinity, temperature, surfactant structure, equilibrium time, and testing methods across various studies. Therefore, the results of this study only verify that the composite system can form an ultralow interfacial tension system under the specific experimental conditions described in this paper. The minimum interfacial tension is observed at a mass fraction of 0.20%, which further indicates that interfacial adsorption basically reaches saturation near this concentration. When the concentration of active components exceeds this value, most of the additional active substances remain in the bulk phase or form aggregates, making it difficult to further improve interfacial properties.

#### 3.1.3 Contact Angle Measurement

As shown in Figure 6, the contact angle of the core increased markedly after soaking in different solutions, indicating that the water-wet core could be effectively altered to a neutral-wet or oil-wet state.

**Figure 6:**
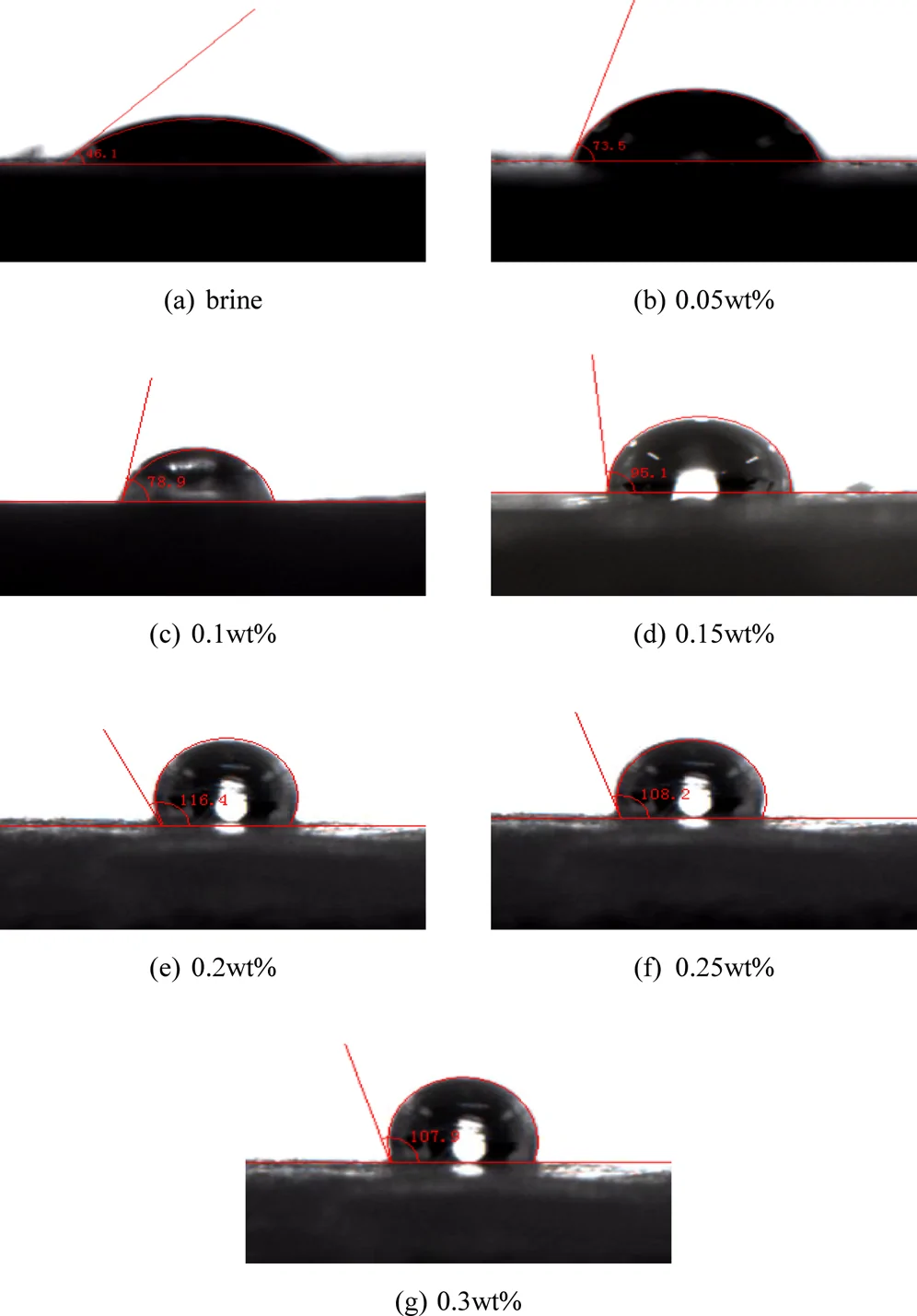
Water contact angles of core slices after aging in nanoemulsion/mud-acid systems at 105 °C for 72 h.

Among the tested systems, the core soaked in the 0.20 wt % nanoemulsion/acid system exhibited the largest contact angle of 116.4°, indicating that the treated surface exhibited a substantially lower affinity for water and an apparent oil-wet tendency under the measurement conditions. This can be attributed to the adsorption of surfactant molecules, which contain hydrophobic tail chains and hydrophilic head groups. The hydrophobic chains are oriented toward the rock surface, while the hydrophilic head groups face the aqueous phase, resulting in enhanced hydrophobicity of the rock surface and making it difficult for the water phase to spread. In particular, perfluorodecyltrimethylsilane (PDTS1) can adsorb and precipitate on the rock surface, forming a hydrophobic layer.

The increase in contact angle measured in this study is consistent with findings from recent relevant research on fluorinated composite systems. The nanosilica/fluorosurfactant system was reported to increase the contact angle of hydrophilic surfaces from approximately 20° to around 130°, while improving the liquid flowback capacity of tight sandstones.^22^ The contact angle measured in this experiment is 116.4°, verifying that the nanoemulsion/mud acid system can markedly reduce the hydrophilicity of treated rock surfaces.

Nevertheless, the favorable direction of wettability alteration depends on the reservoir-fluid system and the displacement process. Although shifting strongly oil-wet sandstone toward intermediate- or weakly water-wet conditions can enhance water imbibition and oil mobilization, mitigating water-lock damage in water-invaded tight gas reservoirs generally requires reducing the rock surface affinity for water and promoting an intermediate gas-wet or water-repellent state.^24^ The seemingly contradictory wettability evolution rules mentioned above indicate that the optimal wettability state depends on reservoir conditions and construction technologies. For the initially water-wet cores adopted in this study, increasing the water contact angle can restrain water film spreading and mitigate capillary retention.

When the concentration further increases to 0.25 and 0.3 wt %, the contact angle shows a slight decrease instead. This is because the adsorption of surfactant molecules on the rock surface reaches saturation at 0.2 wt %. Exceeding this concentration, excess active components form a large number of micelles in the solution, and the surplus surfactant molecules will adsorb on the formed hydrophobic layer in a reverse orientation, forming a bilayer or multilayer structure with hydrophilic groups facing outward. As a result, the hydrophobicity of the rock surface weakens, leading to a reduction in contact angle. Meanwhile, the slightly increased viscosity of the high-concentration system increases the diffusion resistance of active molecules, which is not conducive to the formation of a uniform and dense adsorption layer, and also aggravates this decline trend. This variation law is consistent with the change of interfacial tension, which further confirms that 0.2 wt % is the optimal concentration for wettability alteration of the composite system.

According to the interfacial tension results, the interfacial activity and wettability modification capacity of the system follow the same variation trend with concentration: both increase markedly with the rising dosage of nanoemulsion in the low-concentration range. When the concentration exceeds 0.2 wt %, the growth rate declines substantially due to saturated interfacial adsorption. Meanwhile, higher concentrations will increase the system viscosity and the average size of emulsion droplets, raising the risk of retention and plugging within tiny pore throats of low-permeability reservoirs. Since the 0.2 wt % system can fully satisfy the core requirements of ultralow interfacial tension and effective wettability modification, 0.2 wt % is determined as the optimal concentration for the nanoemulsion/mud acid composite system.

### 3.2 Pore Distribution Characteristics and Determination of the Movable Fluid Boundary

#### 3.2.1 Pore Distribution Characteristics Revealed by CRMI Experiments

Among these parameters, pore radius mainly reflects the size of reservoir storage space, whereas throat radius and its permeability contribution mainly reflect seepage capacity.^25^

As shown in Figure 7, the pore radius distribution peaks of cores 17–2 and 26–1 are located near 135 μm. The frequency of core 17–2 is greater than 2000, while that of core 26–1 is greater than 1000, indicating that this range represents the main concentration area of pore distribution. Both cores exhibit approximately normal distributions. For core 17–2, the high frequency interval is wider, ranging from 105 to 165 μm, with the maximum frequency exceeding 2500; however, its upper limit of pore radius is relatively lower, ranging from 15 to 285 μm. For core 26–1, the high frequency interval is narrower, ranging from 135 to 165 μm, and the overall frequency is lower, less than 1200, whereas the pore radius range is broader, ranging from 15 to 315 μm.

**Figure 7:**
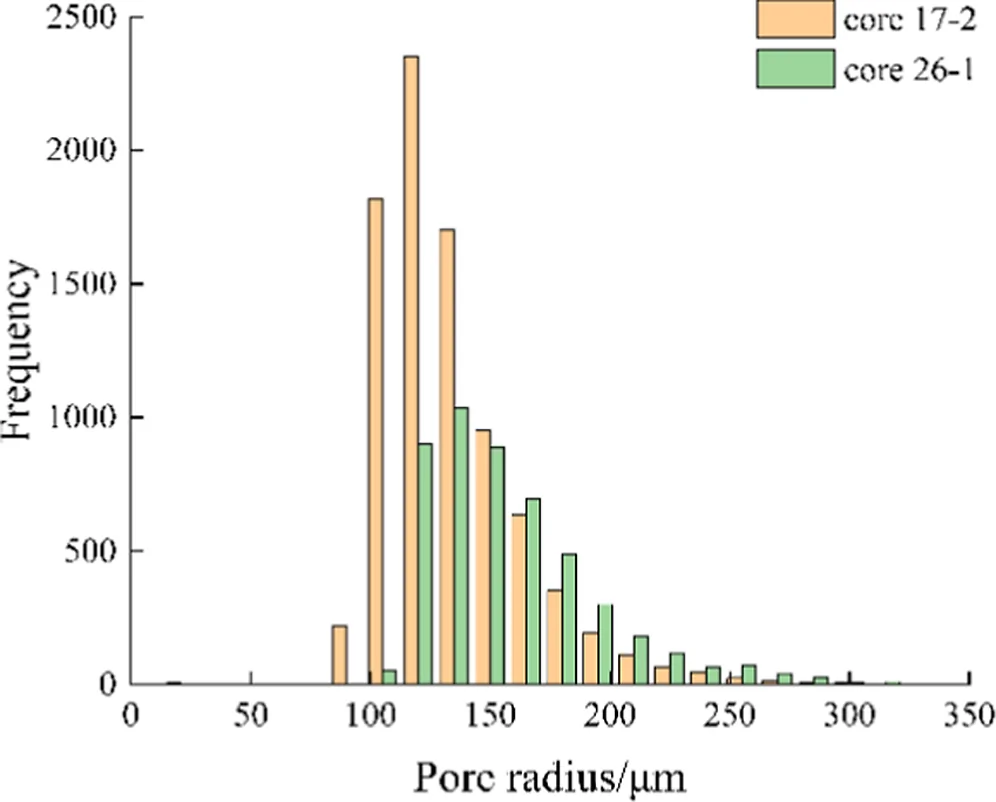
Pore radius distribution characteristics of core 17–2 and core 26–1.

As shown in Figure 8, the permeability contribution of core 17–2 exhibits a bimodal fluctuating distribution, with sharp increases in permeability contribution at pore throat radii of approximately 0.5 and 1.7 μm. This indicates that the internal pore-throat structure of the core is relatively complex, with two dominant seepage channels, while the restriction of small pore throats on fluid flow is significant.

**Figure 8:**
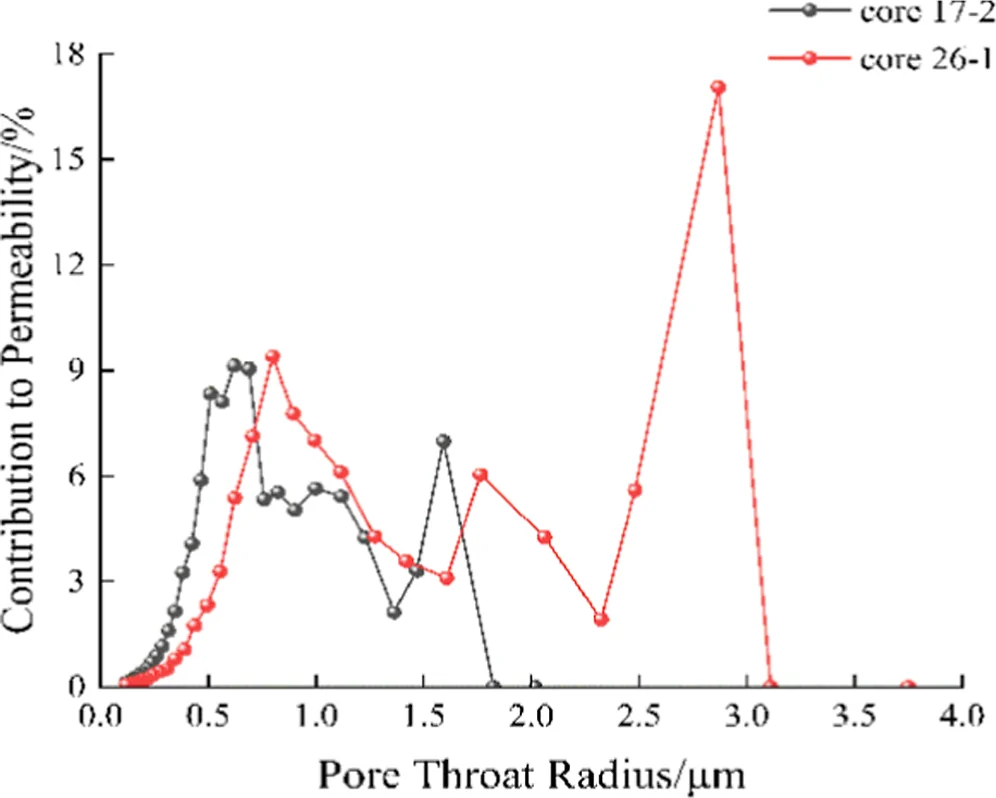
Contribution of pore throat radius to core permeability.

For core 26–1, the permeability contribution shows a unimodal skewed distribution dominated by a single peak. The main peak occurs at approximately 3 μm, and the permeability contribution first increases and then decreases with increasing pore throat radius. This suggests that relatively large throats dominate the overall permeability of core 26–1.

#### 3.2.2 Pore Distribution Characteristics Revealed by NMR Experiments

Nuclear magnetic resonance technology detects hydrogen nuclei in pore fluids, and the acquired signal data reflect the distribution and accumulation of fluids in rocks. The peak area enclosed by signal amplitude and relaxation time corresponds to the amount of fluid contained in the pores. A larger peak area indicates a higher fluid content in the pores, whereas a smaller peak area indicates a lower fluid content.^27^ Pores with relaxation times of 0.01–0.1 ms are classified as micropores, those with relaxation times of 0.1–1 ms are classified as small pores, those with relaxation times of 1–10 ms are classified as medium pores, and those with relaxation times of 10–100 ms are classified as large pores.^28^

Figure 9 shows the *T*_2_ spectra of cores 26–1 and 17–2 after centrifugation at the optimal speed of 10,000 rpm. After centrifugation, formation water in large pores was mobilized to the greatest extent in both cores 26–1 and 17–2, followed by that in medium pores and then small pores, whereas the fluid in micropores was hardly mobilized. According to the Laplace equation, a smaller pore throat radius corresponds to a higher capillary pressure. Therefore, even when a centrifugation speed of 10,000 rpm was applied, corresponding to a high pressure differential, the capillary resistance caused by small pore throats could still not be overcome. This further demonstrates that capillary force is the main mechanism responsible for fluid retention in small pore channels.

**Figure 9:**
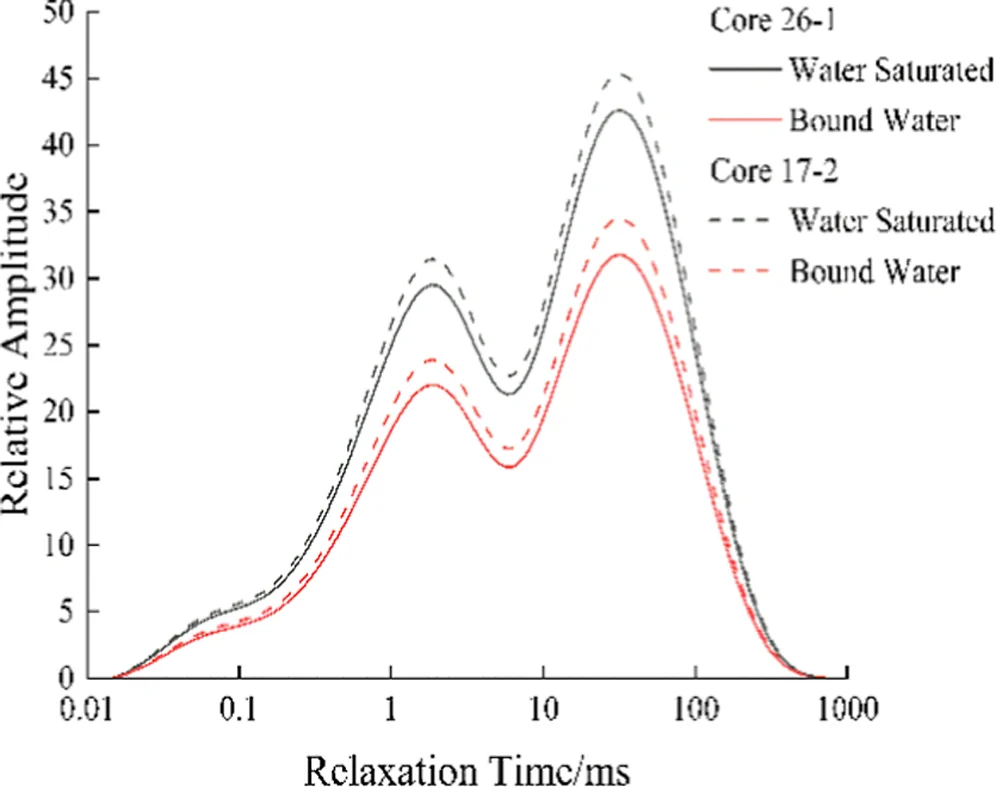
NMR *T*_2_ spectra of Core 26–1 and Core 17–2 after water saturation and centrifugation.

Although the CRMI-derived permeability-contribution distributions of cores 17–2 and 26–1 differ markedly, both NMR spectra exhibit two major relaxation populations. This apparent discrepancy arises because the two techniques are sensitive to different attributes of the pore system. CRMI permeability contribution is strongly controlled by entry throats and connected transport pathways, whereas the NMR *T*_2_ spectrum is primarily weighted by the volume of hydrogen-bearing fluid within pore bodies. Therefore, the two cores may contain broadly similar pore-body fluid populations while differing substantially in the throat networks connecting these pore bodies. The bimodal NMR spectra should consequently not be interpreted as evidence of identical seepage structures.

#### 3.2.3 Determination of the Movable Fluid Boundary

For conversion of the NMR *T*_2_ distribution to an equivalent pore-radius distribution, the fluid-filled pore space was approximated by a cylindrical geometry, for which the pore-shape factor c was set to 2.^18^ This assumption provides a practical equivalent-radius representation rather than a direct reconstruction of the actual pore geometry. To reduce the uncertainty associated with a standalone NMR conversion, the surface relaxivity was calibrated by matching the characteristic CRMI-derived radius distribution with the corresponding *T*_2_ response of the fully water-saturated core. Because CRMI is primarily controlled by pore-entry throats whereas NMR *T*_2_ is weighted by fluid-filled pore volume, the converted NMR radius should be interpreted as an equivalent pore-body-related scale rather than as a direct throat radius. As shown in Figure 10, the increment of mercury saturation varies with pore throat radius and exhibits obvious fluctuations. The relationship between mercury saturation increment and pore-throat radius reflects changes in fluid saturation within the rock pores. Different pore throat radii correspond to different fluid injection or discharge processes, which further leads to variations in mercury saturation increment.

**Figure 10:**
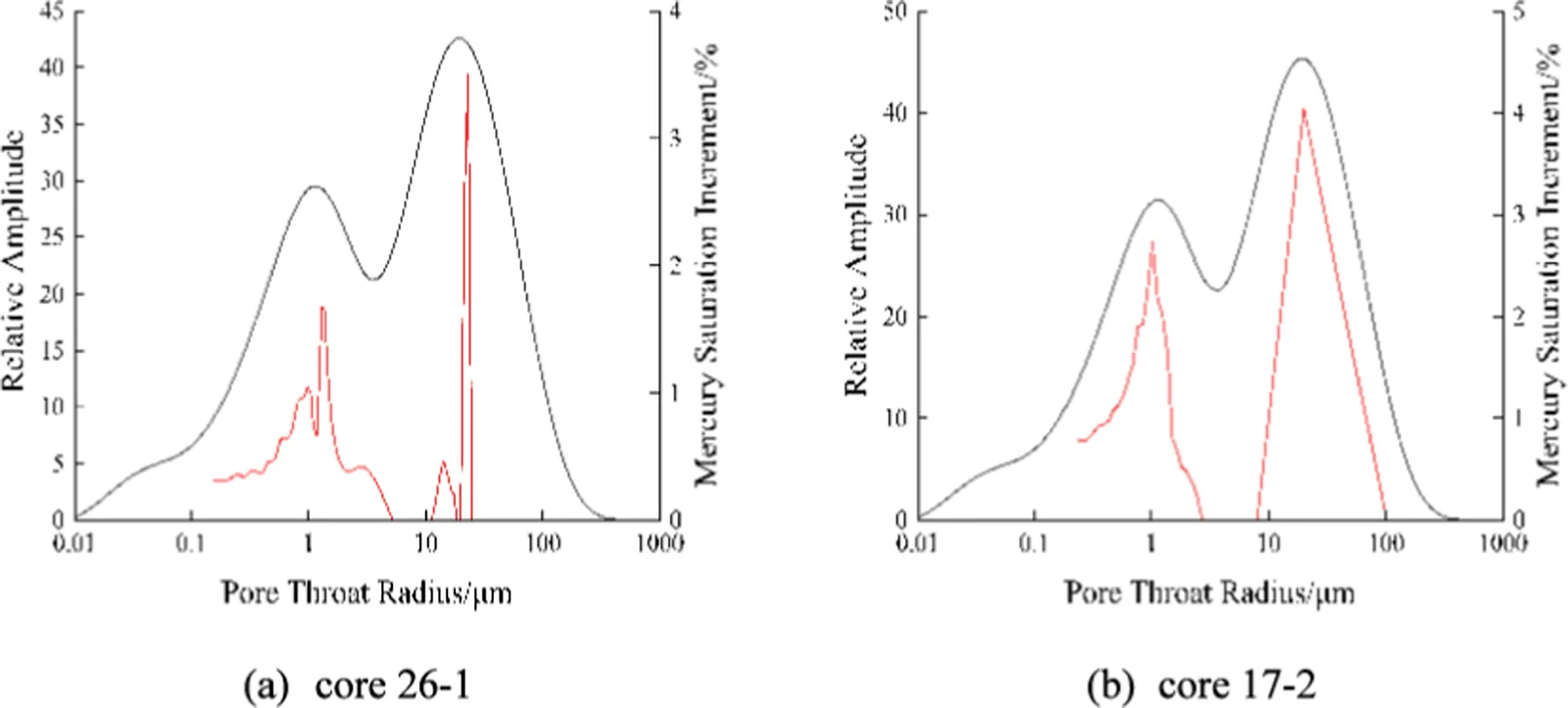
Pore throat radius distribution of core 26–1 and core 17–2 based on combined NMR and CRMI measurements.

Figure 10 further indicates that the mercury saturation increment of core 26–1 is mainly concentrated in small pores, whereas that of core 17–2 is concentrated in small and medium pores, with medium pores being relatively more developed. In addition, the peak of the mercury saturation increment almost coincides with the peak of the relative amplitude.

In previous studies, the lower limit of movable fluid pore throat radius was usually determined by the pore throat radius corresponding to the *T*_2_ cutoff value. However, the experimental results show that when the pore-throat radius is smaller than that corresponding to the *T*_2_ cutoff value, part of the fluid in the core can still be mobilized by centrifugal force. Conversely, when the pore-throat radius is larger than that corresponding to the *T*_2_ cutoff value, bound fluid still exists in the core. Therefore, determining the lower limit of movable fluid pore throat radius solely based on the *T*_2_ cutoff value has certain limitations.

According to the changes in the *T*_2_ spectra before and after centrifugation (Figure 8), the lower limit of movable fluid pore throat radius is not a single value but a range. Accordingly, the fluid occurrence state can be divided into three regions: the bound fluid zone, transition zone, and movable fluid zone. Taking cores 26–1 and 17–2 as examples (Figure 11), the pore throat radius corresponding to the point where the cumulative *T*_2_ curve of the fully water-saturated state begins to separate from that of the bound water state after centrifugation is defined as the boundary between the bound fluid zone and the transition zone, denoted as *r*_a_. When the pore-throat radius is smaller than *r*_a_, the fluid in the pore throats is regarded as bound fluid.

**Figure 11:**
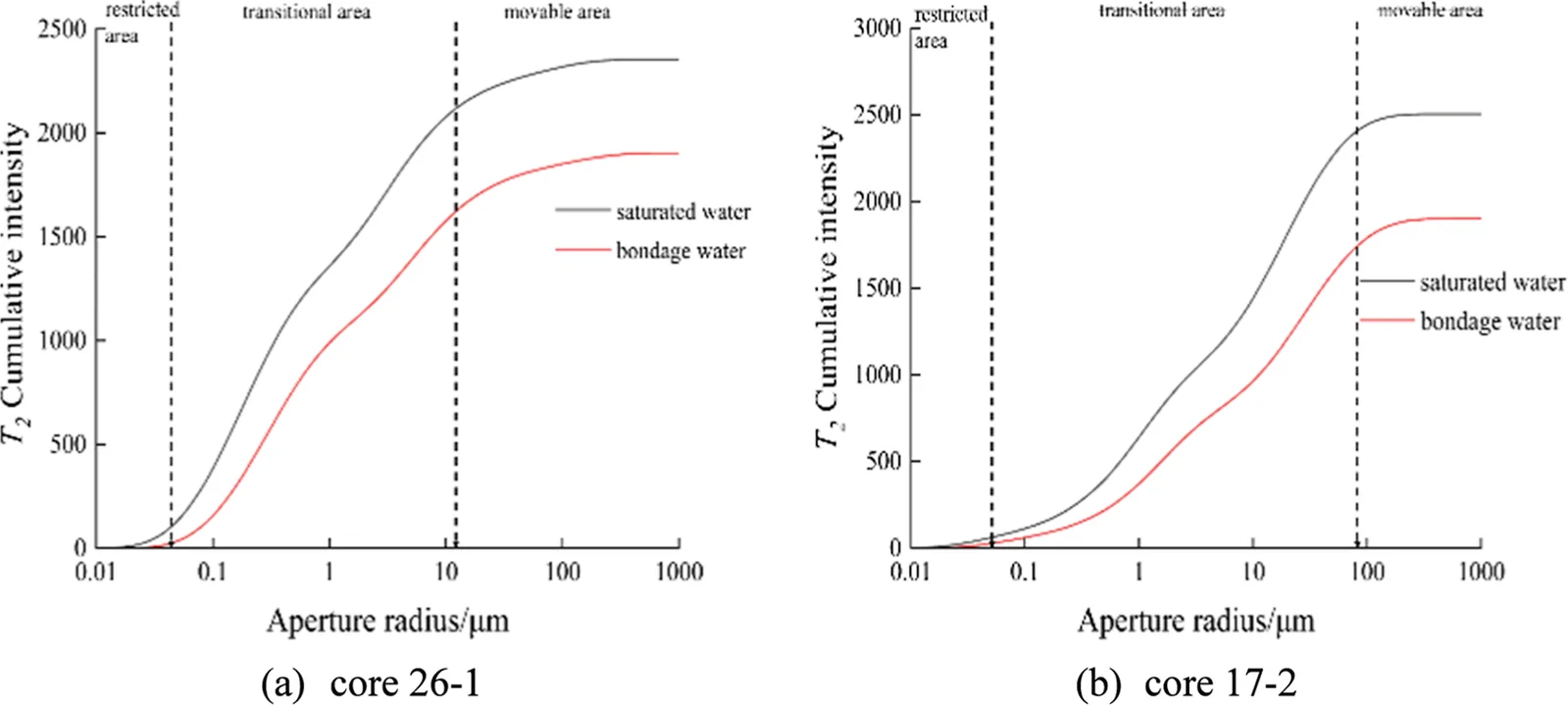
Distribution curves of the lower limit of movable fluids in core 26–1 and core 17–2.

The pore throat radius corresponding to the point where the cumulative intensity of the postcentrifugation cumulative *T*_2_ curve no longer changes is defined as the boundary between the transition zone and the movable fluid zone, denoted as *r*_b_. When the pore throat radius is larger than *r*_b_, the fluid in the pore throats is regarded as movable fluid. When the pore throat radius ranges between *r*_a_ and *r*_b_, part of the fluid in the pore throats is in a bound state, while the remaining part is in a movable state.

As permeability decreases, the core gradually transforms from a pore dominated type to a throat dominated type. Meanwhile, *r*_a_ gradually increases and *r*_b_ gradually decreases. The lower the reservoir permeability, the more the pore-throat structure is characterized by a compressed transition zone range, reduced movable fluid, and increased proportion of bound fluid.

This structural feature directly affects the susceptibility and severity of water locking damage. In reservoirs dominated by small pore throats, with limited movable fluid and a high proportion of bound fluid, capillary resistance becomes significant during water injection or fracturing. As a result, the water phase is more likely to be retained in throats and in large pores controlled by small throats, making it difficult to effectively displace oil and gas and thereby aggravating water locking damage. In contrast, reservoirs with relatively higher permeability contain more movable fluid and better developed seepage channels, allowing the water phase to flow and be discharged more easily; therefore, water locking damage is relatively weaker. Thus, the controlling mechanism of pore-throat structure on movable fluids provides a key basis for understanding and evaluating the microscopic mechanism of water locking damage.

Relevant studies indicate that the lower pore-throat limit of movable fluids does not necessarily correspond to a unique single *T*_2_ cutoff value.^26^ Recent studies of tight sandstones indicate that effective storage volume and accessible pore–throat pathways directly determine the amount of movable fluid, whereas clay-mineral filling, cementation, and pore-network heterogeneity influence fluid mobility by modifying throat dimensions, connectivity, and permeability.^27^

In this study, the fluid occurrence space is further clearly divided into a bound zone, a transition zone, and a movable zone. The transition zone refers to the pore space where movable fluids and retained fluids coexist under the same centrifugal external force. This coexistence phenomenon arises because pores with similar radii are interconnected through throats of varying sizes, thereby exhibiting different capillary trapping capacities. Therefore, the *r*_a_–*r*_b_ interval proposed in this paper should be regarded as a fluid mobility range related to sample properties, rather than a universal pore size threshold.

Based on the three-zone fluid-mobility framework, the water-lock-removal effect of the nanoemulsion/mud-acid composite system can be interpreted as an effective enhancement of fluid mobilization within the transition zone rather than as a directly measured displacement of *r*_a_ and *r*_b_. Mud-acid-induced dissolution may enlarge accessible throats and improve local pore-throat connectivity, thereby reducing the geometric contribution to capillary resistance. Meanwhile, the nanoemulsion reduces oil–water interfacial tension and decreases the affinity of the treated rock surface for water, which facilitates the mobilization of fluids that were previously retained within the transition zone. The greater mobilization of small- and medium-pore fluids observed after composite treatment in the NMR results is consistent with an effective expansion of the movable-fluid domain toward smaller pore sizes. However, because *r*_a_ and *r*_b_ were not independently recalculated after treatment, the present results should not be interpreted as direct quantitative evidence of boundary displacement.

### 3.3 Quantitative Evaluation of Water-Locking Damage

As shown in Figure 12, the water-locking damage coefficients of all four cores increased with increasing pressure differential. However, the overall increasing trend of the water locking damage coefficient of core 11–1 was higher than that of the other three cores. At 30 MPa, the water locking damage coefficient of core 11–1 was 0.93, which was 0.19 higher than that at 5 MPa, where the coefficient was 0.74.

**Figure 12:**
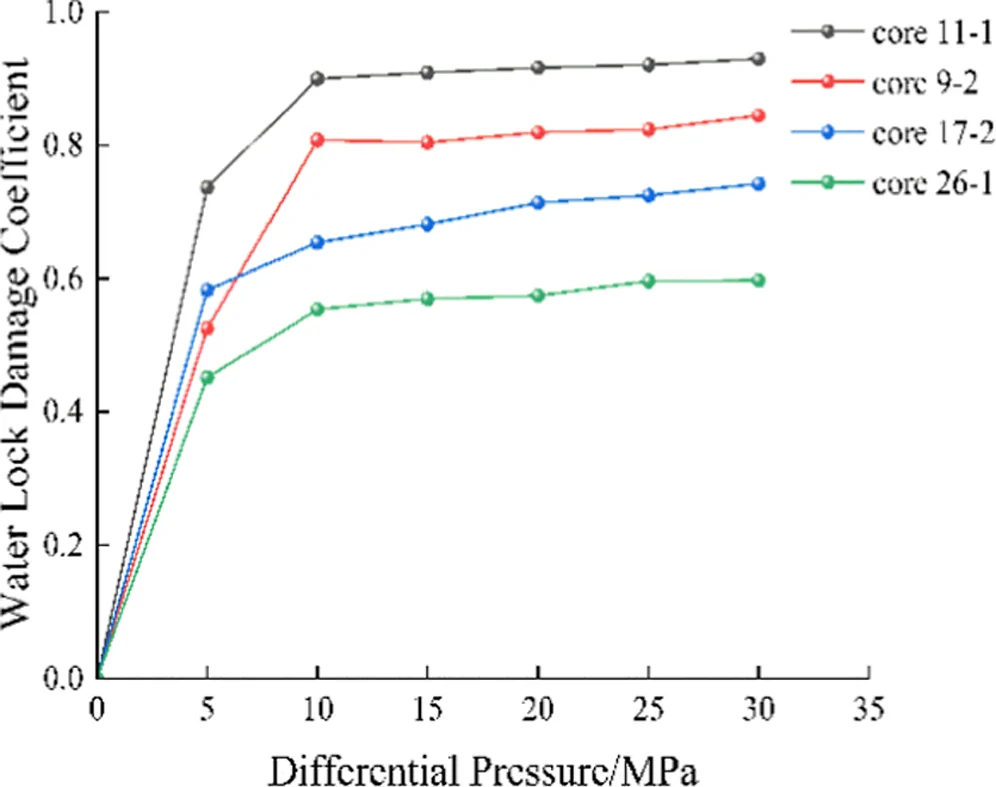
Water-locking damage coefficient (*I*_k_) of different cores as a function of production pressure differential during staged water-invasion experiments.

This difference in damage degree is jointly controlled by permeability, pore-throat structure, water invasion degree and clay mineral sensitivity, and the performance of each core corresponds to its own petrophysical characteristics. Core 11–1, with the highest permeability (3.5 × 10^–3^ μm^2^) and best pore-throat connectivity, suffers the deepest and largest-volume water invasion under the same displacement pressure differential. Although the low-permeability cores exhibited lower overall fluid intake, their finer pore–throat systems imply a greater intrinsic capillary-retention tendency. For the representative low-permeability cores 26–1 and 17–2, the CRMI results reveal fine and/or multiscale throat distributions, which provide a structural basis for stronger capillary resistance according to the Young–Laplace relationship. In addition, the persistence of short-relaxation-time components in the NMR spectra indicates that fluids associated with smaller pore spaces are more difficult to mobilize. These observations support a stronger intrinsic capillary-retention tendency in the low-permeability samples. However, this tendency should be distinguished from the apparent water-lock damage coefficient, which is additionally affected by the total volume and depth of water invasion.

As shown in Figure 13, although the peak values of the *T*_2_ spectra of the four cores differ during displacement, their variations follow a similar pattern. As the production pressure differential increases, crude oil in the pores is gradually displaced. At a pressure differential of 5 MPa, all four cores show limited crude oil production with only minor differences due to the high threshold pressure gradient of low-permeability reservoirs. When the pressure differential rises to 10 MPa, the production gap among cores gradually widens, and core 26–1 produces the largest amount of crude oil, as presented in Figure 13a. Combined with the cumulative oil production curve and the variation of NMR relaxation components, under pressure differentials of 15 and 20 MPa, the growth of cumulative oil production narrows markedly, and only the crude oil signal in large pores corresponding to long relaxation time decreases. Therefore, only a small amount of crude oil in large pores is displaced, while crude oil in small pores is hardly mobilized. Figure 13b shows that core 17–2 produced the highest oil volume at a pressure differential of 5 MPa, followed by that at 10 MPa. Quantitative analysis of the *T*_2_ spectra in Figure 13b shows that at 15 MPa, the signal intensity of the long-relaxation peak (corresponding to large pores) decreases markedly, while the short-relaxation peak (corresponding to small pores) shows almost no attenuation. Therefore, crude oil in small pores is barely displaced, and only crude oil in large pores is produced. Figure 13c shows that, for core 9–2, crude oil in large pores was continuously produced as the production pressure differential increased. However, even under a high pressure differential of 30 MPa, the crude oil in small pores changed only slightly, confirming that capillary force is the main factor responsible for trapping this portion of fluid and is the core cause of water locking damage. Meanwhile, the improvement in oil displacement efficiency became insignificant when the pressure differential exceeded 15 MPa. Figure 13d shows that, with increasing displacement pressure differential, the fluid signals in large, medium, and small pores all decreased systematically. At this stage, the oil phase is redistributed in pore throats. Part of the crude oil in ultrasmall pores first migrates to slightly larger pore throats for temporary storage before being produced, leading to a decrease in the short-relaxation peak amplitude and an increase in the medium-long relaxation main peak amplitude. Meanwhile, the cumulative oil production keeps increasing, and the overall mobilization degree continues to rise.

**Figure 13:**
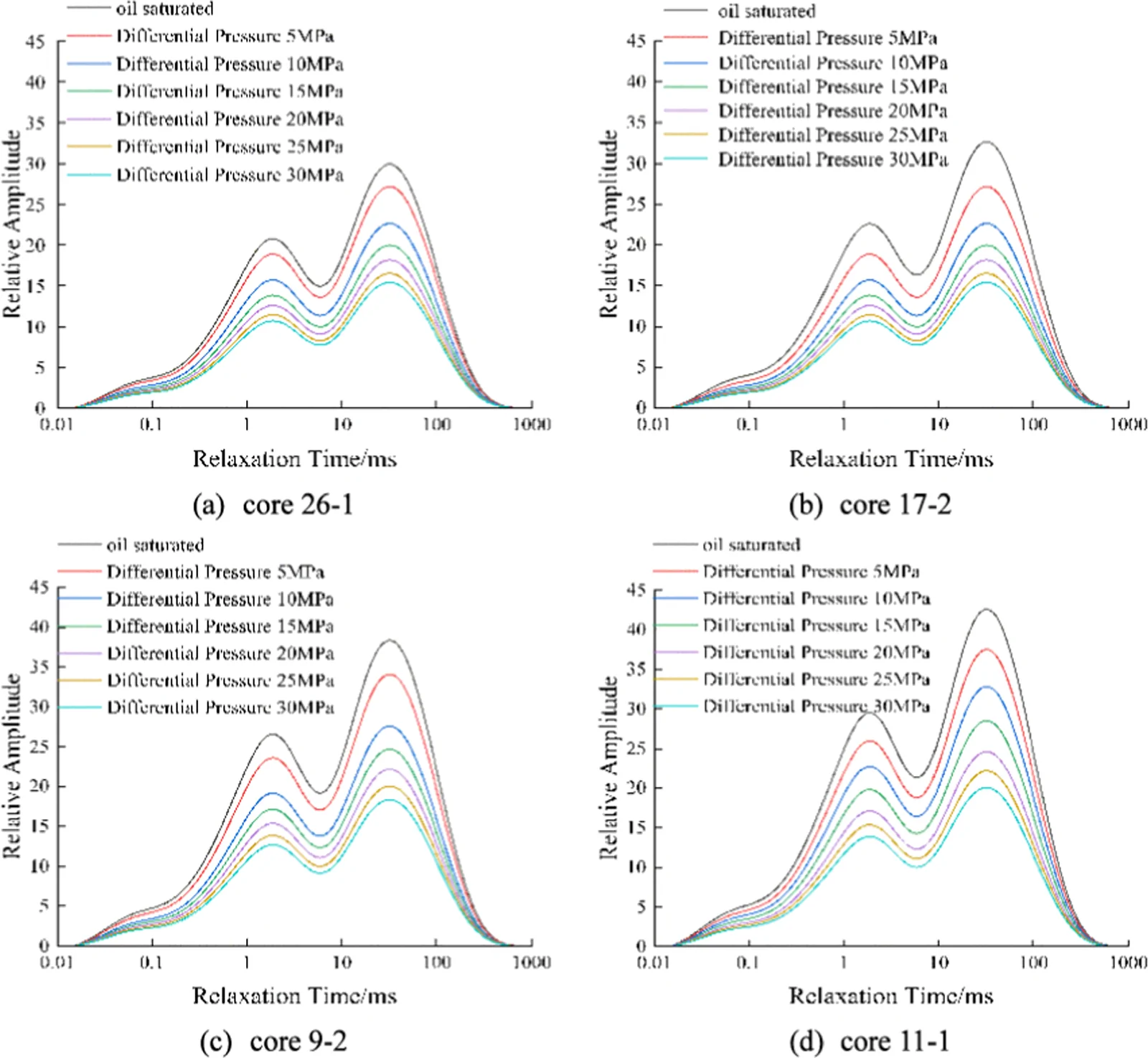
NMR *T*_2_ spectra of different core samples after water flooding under different pressure differences.

Although there are pronounced differences in the pore-throat structure and permeability of the four core samples, all of them develop a two-stage pore system. Hence, the overall variation trend of crude oil mobilization is consistent. Meanwhile, differences exist in the peak positions and the attenuation amplitudes of each relaxation component among different core samples. The pressure gradient required for crude oil mobilization in small pores of low-permeability cores is higher, which reflects the influence of core properties on displacement efficiency.

Therefore, based on the *T*_2_ spectra of the four cores under different production pressure differentials, increasing the production pressure differential can enhance the displacement force and mobilize part of the crude oil in medium and large pores. However, when the pressure differential is excessively high, the water phase rapidly enters dominant flow channels and becomes retained in fine throats and large pores controlled by small throats. As a result, the water phase occupies effective seepage channels, the relative permeability of the oil phase decreases, and the improvement in oil displacement efficiency becomes insignificant, indicating aggravated water locking damage.

Accordingly, the optimal production pressure differential is 10 MPa for core 26–1 and 5 MPa for core 17–2, while the optimal production pressure differential for cores 9–2 and 11–1 is 15 MPa. The NMR *T*_2_ spectra further demonstrate that an excessively high pressure differential aggravates the water locking effect. Therefore, simply increasing the production pressure differential may instead intensify water locking damage.

Recent NMR-based studies of tight sandstones have shown that samples dominated by finer and less-connected pore-throat networks generally exhibit higher bound-water saturation and a stronger tendency for water retention. Their lower permeability can therefore be regarded as an integrated macroscopic manifestation of restricted throat size and connectivity rather than as the sole cause of water retention.^27,28^ However, the relatively high-permeability core 11–1 showed a higher apparent damage coefficient in this study because its better connectivity allowed a larger volume and greater depth of water invasion. Injection-related studies have similarly demonstrated that both the formulation and invasion depth of foreign working fluids influence the severity of formation damage. Polymer residues, flocculent precipitates, retained liquid, and clay hydration or fines migration may obstruct pore-throat flow pathways and consequently reduce sandstone permeability.^29,30^ Therefore, intrinsic capillary trapping should be distinguished from apparent permeability damage controlled by both water retention and the volume and depth of fluid invasion.

However, experimental core 11–1, which possesses the highest initial permeability among all tested samples, exhibits a relatively large water locking damage coefficient. This variation trend differs from the conclusion proposed in several recent studies that permeability is negatively correlated with inherent water locking damage. Such discrepancy originates from differences in experimental methods and displacement processes. The damage coefficient adopted in this paper characterizes the overall reduction in effective permeability after progressive water invasion. Cores with superior connectivity can accommodate more water; under the same sequence of pressure differentials, the aqueous phase penetrates deeper. The invading water occupies connected seepage channels for crude oil and may trigger clay hydration or solid phase plugging, ultimately leading to substantial permeability loss. In contrast, low-permeability cores allow less water ingress, yet the water entering narrow pore throats is trapped by stronger capillary forces.

Therefore, two dimensions of water locking damage need to be distinguished: the inherent fluid retention intensity governed by narrow pore throats, and permeability damage jointly controlled by retention intensity, water invasion volume and water invasion extent. This classification can consistently interpret the experimental observations from this work and relevant recent NMR-based studies, and also demonstrates that the measured damage coefficient cannot be predicted relying merely on permeability parameters.

### 3.4 Evaluation of Water-Locking Removal by the Nanoemulsion/Mud Acid Composite System

As shown in Figure 14, the nanoemulsion/acid system exhibits excellent water-locking removal performance. This is mainly because the nanoemulsion/mud acid system can reduce oil–water interfacial tension and alter rock surface wettability, thereby weakening the spreading and continuous retention capacity of the water phase on the walls of fine pore throats. As a result, the capillary resistance caused by water locking is reduced, and the effective seepage channels for the oil phase are improved. In addition, the mud-acid component can dissolve acid-reactive minerals and may locally reduce geometric restrictions within accessible pore–throat pathways, thereby contributing to improved flow capacity.

**Figure 14:**
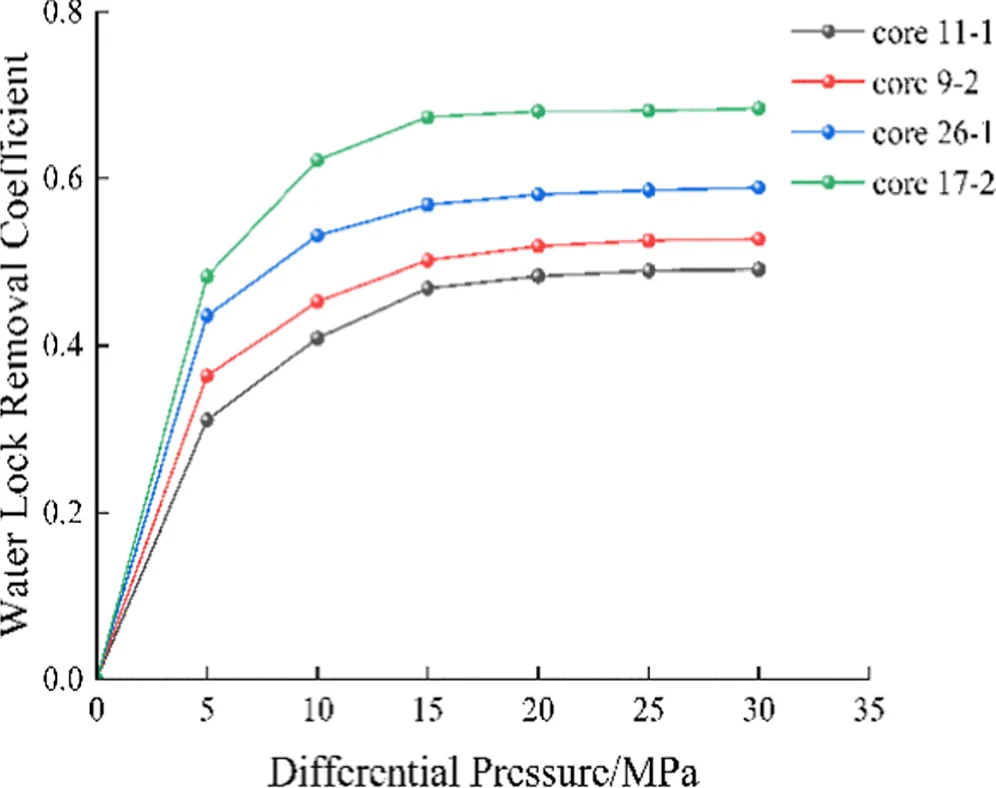
Water-locking recovery coefficient (*I*_a_) of different cores after treatment with the 0.20 wt % nanoemulsion/mud-acid composite system at different production pressure differentials.

The water locking recovery coefficient increases markedly with increasing pressure differential. This is because, although the combined effect of ultralow interfacial tension and acid treatment reduces capillary resistance, relatively large capillary resistance may persist in the smallest or poorly connected pore-throat domains. Therefore, a higher pressure gradient is required to overcome capillary resistance and displace the mobilized crude oil. When the displacement pressure is controlled within the range of 15–20 MPa, a relatively high recovery efficiency can be achieved, with a recovery coefficient of at least 0.35, while avoiding the fracture risk associated with excessive pressurization.

Recent studies have reported substantial permeability recovery after fluorinated composite treatment of tight sandstone, demonstrating that wettability modification can improve aqueous-phase flowback and restore effective flow capacity.^22^ The present results similarly show a marked increase in flow capacity after treatment with the nanoemulsion/mud-acid composite system. However, the numerical recovery coefficients should not be directly compared because the definitions differ among studies. In the present work, $I_{a}=\frac{K_{m}-K_{f}}{K_{m}}$ quantifies the proportion of restored permeability relative to the post-treatment permeability, whereas some previous studies define permeability recovery as the ratio of post-treatment permeability to initial permeability.

The mechanism identified in this study also differs from that of a purely wettability-modifying system. In addition to decreasing water affinity and oil/water interfacial tension, the mud-acid component can dissolve acid-reactive minerals and cements. The permeability response therefore reflects a coupled interfacial and structural effect. The gradual plateau of *I*_a_ above approximately 15–20 MPa suggests that, once the readily accessible oil and water have been mobilized, further pressure increase provides limited additional benefit because fluids remaining in the smallest or poorly connected pore throats remain capillary-bound.

The mineral composition and content of core samples were tested by means of X-ray diffraction (XRD) to clarify the effect of acidizing plug removal. D8 AA25 diffractometer manufactured by Bruker Corporation of Germany was adopted for the test, equipped with a Cu-target ceramic X-ray tube with a maximum power of 3 kW and a focal spot size of 0.4 × 12 mm. The test temperature was 25 °C, the humidity was 40%, and the corresponding results are shown in Table 3. By comparing the content changes of different mineral components before and after water-locking removal, the interactions between the acid system and clay minerals and their effects on pore-throat structure and seepage capacity were investigated. The reaction equations between different mineral components and the acid system are expressed in Appendix (eqs 4–11).

**Table 3:** Mineral Composition before and after Water-Locking Removal

		whole rock content (%)				
no	remarks	quartz	feldspar	calcite	dolomite	clay minerals
11–1	before experiment	31.5	27	17.8	16.9	6.8
	after experiment	35.7	27.5	14.4	15.2	7.2
9–2	before experiment	39.5	15.7	15.6	17.5	11.7
	after experiment	44.1	15.7	12.4	15.5	12.3
26–1	before experiment	35.8	24.6	12.8	14.6	12.2
	after experiment	35.8	24.5	10.1	12.9	12.7
17–2	before experiment	33.6	18.9	14.8	20.8	11.9
	after experiment	37.8	19.1	11.9	18.6	12.6

As shown in Table 3, the relative chlorite content decreases after treatment, whereas the relative proportion of kaolinite increases. The decrease in chlorite is consistent with preferential acid attack on chlorite-bearing phases, which are relatively susceptible to dissolution under strongly acidic conditions. However, the apparent increase in kaolinite should not be interpreted as direct evidence of chlorite-to-kaolinite transformation. Because the XRD results represent normalized relative mineral abundances, dissolution of carbonates, chlorite, and other acid-reactive phases may lead to relative enrichment of the remaining mineral components without requiring their absolute formation. Therefore, the observed mineralogical variation is interpreted primarily as selective dissolution and relative enrichment rather than as direct mineral transformation.

HF reactions with silicate minerals may also generate fluorosilicate species, and subsequent dilution or an increase in pH can potentially promote secondary silica precipitation. Such precipitation represents a possible source of secondary pore–throat blockage during mud-acid treatment. However, secondary silica was not directly quantified in the present experiments; therefore, it is discussed here as a potential acidizing risk rather than as an experimentally confirmed process.

The water-lock-removal response of the composite formulation is interpreted as a coupled interfacial and mineral-modification process. The nanoemulsion component provides interfacial regulation by reducing oil–water interfacial tension and decreasing the affinity of the treated sandstone surface for water, as directly supported by the interfacial-tension and contact-angle measurements. In parallel, the mud-acid component reacts with acid-sensitive minerals, as indicated by the measured mineral-composition changes, and may locally modify accessible pore–throat surfaces. These two processes act on different contributors to water-lock damage: interfacial regulation reduces capillary resistance, whereas mineral dissolution may reduce geometric restrictions within accessible flow pathways. The observed permeability recovery and enhanced NMR fluid mobilization after treatment are consistent with this coupled interpretation. However, because separate nanoemulsion-only and mud-acid-only control experiments were not performed, the present results do not quantitatively establish a greater-than-additive combined effect.

Figure 15 shows representative scanning electron microscope (SEM) morphological characteristics of four groups of processed core samples. Irregular particle surfaces, loose fine particles, sheet-like clay minerals and partial pore structures can be observed. Core samples 9–2 and 17–2 contain abundant fine particles and clay mineral fillings, while core samples 26–1 and 11–1 are dominated by primary micropore throats. Clay minerals are mostly attached to pore walls, resulting in a denser overall pore structure.

**Figure 15:**
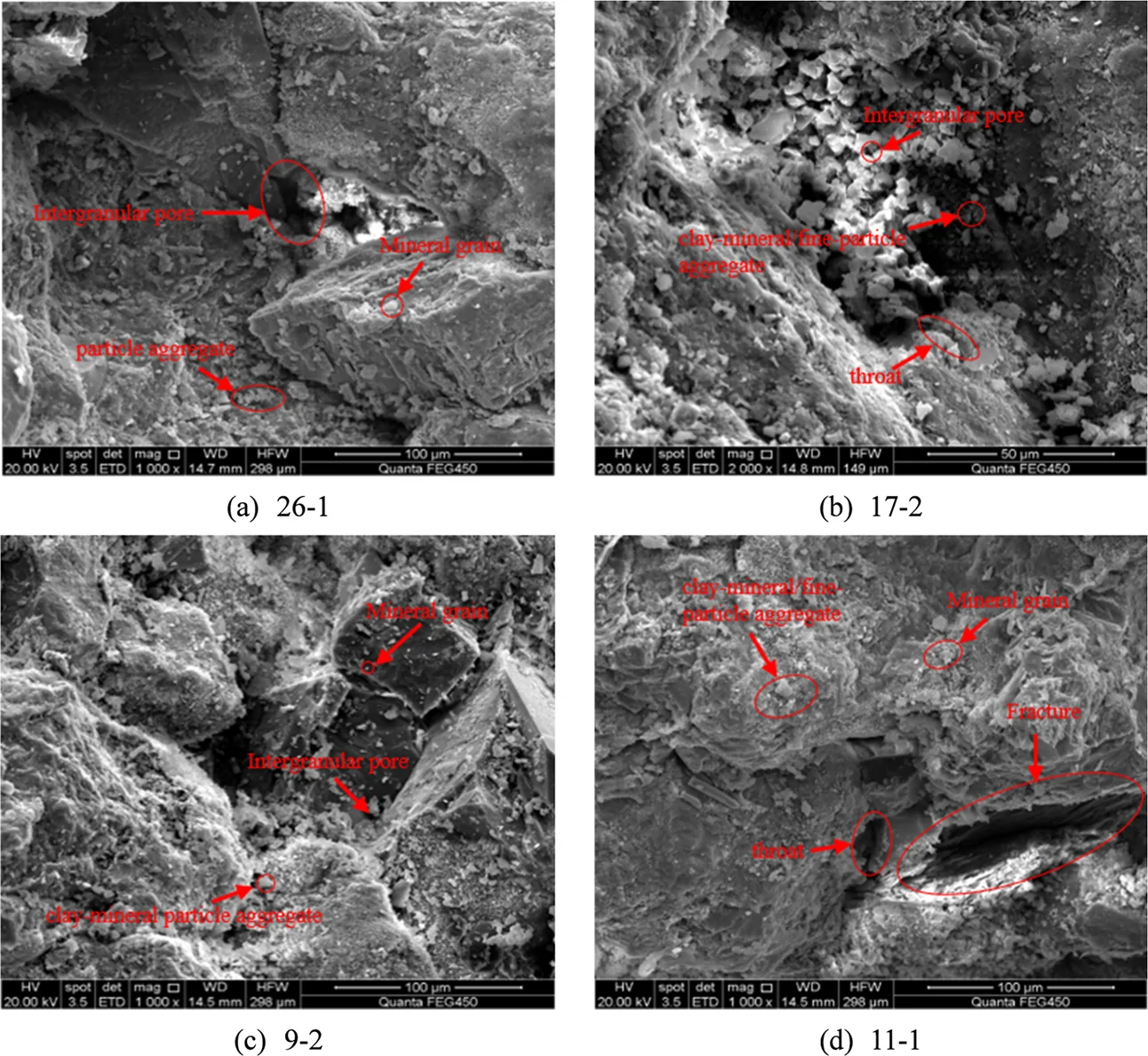
Representative post-treatment SEM images of cores 26–1, 17–2, 9–2, and 11–1 after nanoemulsion/mud-acid treatment.

However, due to the lack of untreated control images captured at the same observation positions and magnification, the available SEM images alone cannot definitively verify the generation of new pores or the overall improvement of pore-throat connectivity. Accordingly, the above images are only regarded as qualitative evidence for surface modification and partial mineral dissolution of cores after treatment. The improvement of effective flow channels is mainly judged based on variations in mineral composition. The above observations comprehensively indicate that the nanoemulsion-mud acid system can induce partial modification of the pore-throat structure, by integrating CRMI pore structure data, XRD mineral analysis and permeability test results, it is concluded that the modification performance of the composite system is governed by the original pore-throat structure and reservoir heterogeneity. Cores with larger initial throat radii and lower clay filling exhibit more uniform dissolution and higher permeability recovery. Meanwhile, microscale heterogeneity in pore structure and mineral occurrence may result in spatially variable acid-induced modification among different cores and fields of view, consistent with the nonuniform mineral dissolution and pore–throat alteration reported for acidized tight sandstones.^17^

Recent multiscale studies of tight sandstones have demonstrated that fluid mobility and permeability should be interpreted by jointly considering mineral composition and diagenetic cementation or filling, pore–throat geometry and connectivity, and the pore-scale distribution of bound and movable fluids.^27,31^ This supports the comprehensive analytical approach adopted in this study. After acidification treatment, the relative contents of calcite and dolomite decrease, which is consistent with the rule that carbonate minerals preferentially react with acidic components. The relative proportions of quartz and some clay minerals increase accordingly, which cannot be simply interpreted as an absolute growth in the production of such minerals. This is because mineral proportions measured by X-ray diffraction are normalized closed-sum data; the observed increase in proportions partly arises from the relative enrichment effect after the dissolution of acid-soluble components.

The post-treatment SEM images exhibit irregular mineral surfaces, locally exposed pore spaces, and morphological features consistent with partial mineral dissolution. Because corresponding untreated images from identical locations were unavailable, these observations cannot independently demonstrate throat enlargement or improved pore connectivity. When considered together with the XRD mineral-composition changes, NMR fluid-mobilization response, and permeability recovery, the SEM observations provide qualitative support for localized pore–throat modification after treatment.

As shown in Figure 16, the composite treatment mobilized more crude oil than water flooding under the tested conditions, including part of the fluid associated with smaller pore domains as the pressure differential increased. Reduction of oil–water interfacial tension and capillary resistance is an established fundamental function of surfactant-based treatments and is therefore regarded here as a basic working mechanism rather than as a unique contribution of the present formulation. Compared with purely surfactant-based treatments, the present nanoemulsion/mud-acid composite system addresses a broader range of factors contributing to water-lock damage. The distinction between the present nanoemulsion/mud-acid composite formulation and a purely surfactant-based treatment lies primarily in the range of water-lock-controlling processes addressed rather than in a universally superior interfacial performance. During the long-term aging test, the mean droplet size changed from 35.7 to 39.8 nm under the tested high-temperature and high-salinity conditions, indicating limited droplet growth and acceptable colloidal stability. Separately, the fresh composite formulation achieved ultralow oil–water interfacial tension and substantially reduced the water affinity of the treated sandstone surface. In addition to these interfacial responses, the mud-acid component was associated with measurable changes in acid-reactive mineral contents, whereas the NMR and permeability-recovery results indicated enhanced fluid mobilization after treatment. Therefore, the main feature of the composite treatment is that interfacial regulation and local mineral modification are addressed within the same formulation and evaluated within a common pore-scale framework. Because the different formulations reported in previous studies were tested under different oil compositions, temperatures, salinities, and experimental protocols, the present results should not be interpreted as evidence of universal superiority over conventional surfactant systems.

**Figure 16:**
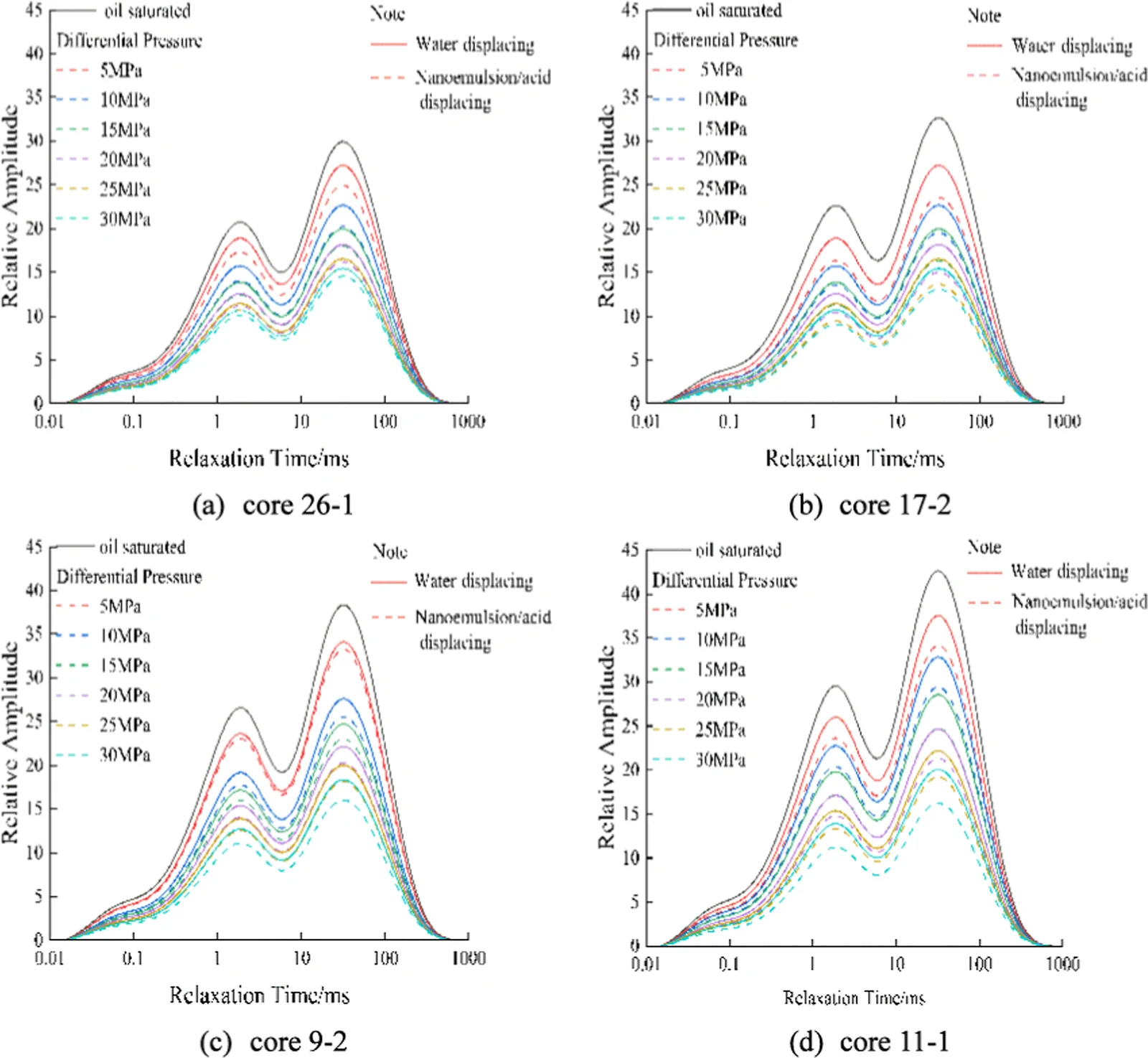
Comparison of NMR *T*_2_ spectra before and after nanoemulsion/mud-acid treatment, showing changes in fluid mobilization within different pore-size ranges.

## 4 Conclusions

1. This study establishes an integrated framework combining CRMI, NMR, and core-flooding experiments to characterize pore-throat controlled fluid mobility and water-lock damage in low-permeability sandstone. The results indicate that the movable-fluid boundary is better represented by a sample-dependent transition interval than by a single *T*_2_ cutoff or pore-size threshold; accordingly, the fluid-occurrence space is divided into bound-fluid, transition, and movable-fluid zones.
2. Production pressure differential has a dual effect on fluid mobilization. Increasing pressure initially strengthens displacement, whereas excessive pressure promotes deeper aqueous invasion and occupation of effective oil-flow pathways. Water-lock damage is therefore controlled by both intrinsic capillary trapping and the volume and depth of water invasion.
3. At a concentration of 0.20 wt %, the nanoemulsion/mud-acid composite system achieved an oil–water interfacial tension of 0.486 × 10^–3^ mN·m^–1^ and an apparent static water contact angle of 116.4°, indicating ultralow interfacial tension and reduced water affinity of the treated sandstone surface. Combined mineralogical, NMR, and permeability-recovery results indicate that water-lock removal is associated with coupled interfacial regulation and localized mineral dissolution.
4. Post-treatment SEM observations provide qualitative evidence of localized surface alteration but do not independently demonstrate new-pore generation or overall connectivity enhancement. Together with the XRD, NMR, and permeability-recovery results, these observations support an interpretation in which interfacial regulation and localized mineral dissolution jointly facilitate fluid mobilization within accessible pore–throat pathways.
5. The contribution of this study lies in identifying that the transition zone is the pore size interval where water blocking damage and its recovery are most significant, which provides a mechanistic basis for collaborative optimization and production pressure differential. Nevertheless, the mechanism proposed in this paper and field working conditions still need to be verified by more core experiments and field tests.

## 5 Limitations and Outlook

1. This study is based on four core samples, and the CRMI-NMR mobility-zone model was established primarily using two representative samples. Therefore, the proposed mobility boundaries are sample-dependent and should not be regarded as universal thresholds. The local slope transition was not verified using independent interfacial-rheology measurements or sufficient replicate kinetic tests. The mobility boundaries derived from cores 26–1 and 17–2 are sample-specific and should not be interpreted as universal thresholds for the entire reservoir. In addition, contact angles were measured at room temperature, and the post-treatment SEM images were not obtained from matched pre- and post-treatment locations. The SEM observations consequently provide qualitative rather than quantitative evidence of pore-structure modification. The study also lacks field-scale data on long-term injectivity, corrosion, chemical retention, flowback treatment, economic performance, and environmental effects. This study lacks Atomic Force Microscopy tests and 3D profilometer characterizations, making it impossible to distinguish the respective contributions of chemical properties and surface morphology to wettability. Follow-up research will combine Atomic Force Microscopy roughness measurements and contact angle experiments under reservoir conditions.
2. Future work should expand the number of core samples and include parallel experiments for reservoirs with different permeability and clay-mineral contents. In situ contact-angle and interfacial measurements under reservoir pressure and temperature are needed to evaluate the actual wettability response. Micro-CT, SEM, CRMI, and NMR measurements should be combined to quantify three-dimensional pore-throat modification and possible secondary precipitation. Field pilot tests should further evaluate injectivity, long-term permeability recovery, corrosion, chemical retention, flowback-water treatment, environmental risk, and techno-economic feasibility.

## Appendix

The models and manufacturers of the experimental equipment used in this study are summarized in Table 4.

**Table 4:** Model and Manufacturer of Experimental Equipment

no	name	model	manufacturer
1	high-temperature and high-pressure multifunctional core flooding experimental system	HBYQ-2	Yangzhou Huabao Petroleum Instrument Co., Ltd.
2	electronic analytical balance	LE-104E	Ningbo Zhenzhou Huafeng Instrument Factory
3	video rotating drop tensiometer	SVT-20N	Beijing Weihui Jiake Technology Co., Ltd.
4	constant temperature water bath	DHC-05-AW	Hangzhou Qiwei Instrument Co., Ltd.
5	electric hot air oven	DHG-9070(A)	Shanghai Yiheng Scientific Instrument Co., Ltd.
6	immersion-type constant temperature magnetic stirrer	DF-101S	Shanghai Licheng Bixi Instrument Technology Co., Ltd.
7	desktop high-speed centrifuge	LC-LX-H185C	Shanghai Licheng Bixi Instrument Technology Co., Ltd.
8	constant-rate mercury porosimeter	ASPE-730	Coretest Systems, USA
9	nuclear magnetic resonance spectrometer	SPEC-RC2	Beijing Sipaike Science & Technology Development Co., Ltd.
10	Malvern Mastersizer laser particle size analyzer	Mastersizer 2000	Zhuhai OMEC Instruments Co., Ltd.
11	Bruker X-ray diffractometer	D8AA25	Bruker AXS GmbH

Reaction equations for mineral–acid interactions

4
$${calcite:\;CaCO}_{3}+{2HCl\to CaCl}_{2}+{CO}_{2}+H_{2}O$$

5
$$dolomite:\;CaMg{\left({CO}_{3}\right)}_{2}+{4HCl\to CaCl}_{2}+{MgCl}_{2}+{2CO}_{2}+{2H}_{2}O$$

6
$${quartz:\;SiO}_{2}+{4HF\to SiF}_{4}+{2H}_{2}O$$

7
$${albite:\;NaAlSi}_{3}O_{8}+{22HF\to NaF+AlF}_{3}+{3H}_{2}{SiF}_{6}+{8H}_{2}O$$

8
$${K‐feldspar:\;KAlSi}_{3}O_{8}+{22HF\to KF+AlF}_{3}+{3H}_{2}{SiF}_{6}+{8H}_{2}O$$

9
$${kaolinite:\;Al}_{2}{Si}_{2}O_{5}{\left(OH\right)}_{4}+{18HF\to 2AlF}_{3}+{2H}_{2}{SiF}_{6}+{9H}_{2}O$$

10
$${chlorite:\;Mg}_{5}{Al}_{2}{Si}_{3}O_{10}{\left(OH\right)}_{8}+{16HCl\to 5MgCl}_{2}+{2AlCl}_{3}+{3H}_{4}{SiO}_{4}+{6H}_{2}O$$

11
$${illite:\;KAl}_{3}{Si}_{3}O_{10}{\left(OH\right)}_{2}+{28HF\to KF+3AlF}_{3}+{3H}_{2}{SiF}_{6}+{12H}_{2}O$$

## Abbreviation and Symbol Explanation

CRMI: constant-rate mercury intrusion
NMR: nuclear magnetic resonance
SEM: scanning electron microscopy
XRD: X-ray diffraction
IFT: interfacial tension
PDTS1: verified chemical name of PDTS1 (perfluorodecyltrimethylsilane)
JFC: nonionic penetrant JFC
D_2_O: heavy water
*T*_2_: transverse relaxation time
*I*_k_: water-locking damage coefficient
*I*_a_: water-locking recovery coefficient
*K*_D_: permeability of the cleaned and dried core
*K*_f_: permeability after water-locking damage
*K*_m_: permeability after water-lock remediation
ρ: surface relaxivity
*V*/*S*: pore-volume-to-surface-area ratio
*c*: pore-shape factor
*r*: pore radius

## References

[1] Wang L. H., Li X. F., Wang J. J., Zhang H. B., Shi H. G., Liu G. F., Yang D. Y. (2025). Effect of Pore-Throat Structure on
Irreducible Water Saturation and Gas Seepage Capacity in a Multilayer
Tight Sandstone Gas Reservoir. Geoenergy Sci.
Eng..

[2] Qiang X., Wen K., Wang D., Fan H., Jia L., Yang G., D J., Shen X., Yao H. (2025). Differences
in Pore-Throat Structure
and Seepage Characteristics of Tight Sandstone Gas Reservoirs and
Their Effects on Water-Blocking: A Case Study of the Shanxi, Taiyuan,
and Shihezi Formations in the Shenmu Gas Field, Ordos Basin. J. Northeast Pet. Univ..

[3] Tang H. M., Zhu B. Y., Wang X., Zhang L. H., Zhao F., Wang J. J. (2018). Mechanism and Control
Factors of Water Blocking in
Tight Sandstone Gas Reservoirs. Sci. China:
Technol. Sci..

[4] Wang J., Li Y. F., Zhou F. J., Yao E. D., Zhang L., Yang H. (2020). Study on the Mechanism
of Nanoemulsion Removal of Water Locking Damage
and Compatibility of Working Fluids in Tight Sandstone Reservoirs. ACS Omega.

[5] Sun Z. H., Li M. H., Yuan S., Hou X. Y., Bai H., Zhou F. J., Liu X. F., Yang M. M. (2024). The Flooding Mechanism
and Oil Recovery of Nanoemulsion on Fractured/Non-Fractured Tight
Sandstone Based on Online LF-NMR Experiments. Energy.

[6] Dabiri A., Honarvar B. (2020). Investigation of Interfacial
Tension Reduction, Wettability
Alteration, and Oil Recovery Using a New Non-Ionic Oil-Based Surfactant
from the Gemini Surfactants Family Coupled with Low-Salinity Water:
Experimental Study on Oil-Wet Carbonate Rock. J. Surfactants Deterg..

[7] Shafiq M. U., Chong Y. J., Mahmud H. K. B., Hossain M. M., Rezaee R., Testamanti N. (2019). Application of Emulsified Acids on Sandstone Formation
at Elevated Temperature Conditions: An Experimental Study. J. Pet. Explor. Prod. Technol..

[8] Wei Z. Y., Dai C. L., Jiang W. D., Zhang J., Yang S., Zhao J. (2015). Screening and Evaluation of an Acidification
Pre-Plug-Removal System
for an Offshore Reservoir. Oilfield Chem..

[9] Fu W. Y., Zheng J. G., Zheng Y. C., Yin Z. (2018). Research and Application
of a Retarded Acidizing and Plug-Removal System in the Chang 3 Reservoir. J. Yangtze Univ. Nat. Sci. Ed..

[10] Jia W., Xian C., Jia B., Wu J. (2023). A High-Temperature
Retarded Acid Based on Self-Assembly of Hydrophobically Associating
Polymer and Surfactant. J. Mol. Liq..

[11] Mahmoud M., Aljawad M. S., Kamal M. S., Shakil
Hussain S. M. (2020). Sandstone
Acidizing Using a New Retarded Acid System Based on Gemini Surfactants. J. Pet. Sci. Eng..

[12] Bai H., Zhang K., Zhou F. J., Yao E. D. (2024). Study on Combination
of Surfactant and Acid for Depressure and Increasing Injection Oil
Displacement in Heterogeneous Sandstone Reservoirs. Phys. Fluids.

[13] Zhang Q. P., Liu Y. C., Wang B. T., Ruan J. F., Yan N., Chen H., Wang Q., Jia G. W., Wang R. N., Liu H., Xue C. W., Liu F. L., Yang H., Zhu Y. S. (2022). Effects
of Pore-Throat Structures on Fluid Mobility in Chang 7 Tight Sandstone
Reservoirs of the Longdong Area, Ordos Basin. Mar. Pet. Geol..

[14] Meng Z. Y., Sun W., Liu Y. Q., Luo B., Zhao M. R. (2021). Effect of Pore Networks
on the Properties of Movable Fluids in Tight Sandstones from the Perspective
of Multiple Techniques. J. Pet. Sci. Eng..

[15] Hong L. V., Mahmud H. B. (2019). A Preliminary Screening and Characterization
of Suitable
Acids for Sandstone Matrix Acidizing: A Comprehensive Review. J. Pet. Explor. Prod. Technol..

[16] Wang J., Huang Y. X., Zhou F. J., Song Z. Y., Liang X. Y. (2020). Study on
Reservoir Damage during Acidizing for High-Temperature and Ultra-Deep
Tight Sandstone. J. Pet. Sci. Eng..

[17] Wang X. W., Yang Z. M., Li H. B., Guo H. K. (2010). Method
for Studying
the Pore Structure of Low-Permeability Reservoirs Using Nuclear Magnetic
Resonance. J. Southwest Pet. Univ..

[18] Zhang J. G., Wang M., Chu Y. P., Wang M. J., Song S. Q., Zheng C., Liu F., Ouyang K., Wang X. L., Zhang X., Xu W. L. (2026). Experimental
Study on the Influence
of High-Temperature Treatment on the Pore Structure and Energy Evolution
Characteristics of Sandstone. ACS Omega.

[19] Handa M., Ujjwal R. R., Vasdev N., Flora S. J. S., Shukla R. (2021). Optimization
of Surfactant- and Cosurfactant-Aided Pine Oil Nanoemulsions by Isothermal
Low-Energy Methods for Anticholinesterase Activity. ACS Omega.

[20] Maji N. C., Rastogi P., Krishnasamy A., Aidhen I. S., Kaisare N. S., Basavaraj M. G. (2022). Storage
and Temperature Stability of Emulsified Biodiesel-Diesel
Blends. ACS Omega.

[21] Zhang Y., Zhang D. X., Wang Y., Chen J., Xin M. K., Zhang Q. R., Qin Y. W., Yan L. S. (2025). Experimental
Study
on a Nanoemulsion for EOR in High-Temperature, High-Salinity, Low-Permeability
Reservoirs and Its Performance. ACS Omega.

[22] Chen C., Zhuo S. Q., Li S. Z., Yin N. X., Luo C., Ren H., Jia M., Wang X. Y., Cheng Q. (2025). Wettability Modification
of a Nano-Silica/Fluorosurfactant Composite System for Reducing Water-Blocking
Damage in Tight Sandstone Reservoirs. RSC Adv..

[23] Shen Y., Wu J. C., Shi F., He Y. W., Li H. J., Zhou W. X., Zhao M. Y., Wang C. Y., Yan Y., Lu M. Z., Ma J. S., Zheng L. L. (2023). Study on Green Nanofluid
Profile Control and Displacement of Oil in a Low-Permeability Reservoir. ACS Omega.

[24] Alajmei S. (2023). Wettability
Alteration of Berea Sandstone for Gas Condensate Applications. ACS Omega.

[25] Xiao D. S., Lu S. F., Lu Z. Y., Huang W. B., Gu M. W. (2016). Combining
Nuclear Magnetic Resonance and Rate-Controlled Porosimetry to Probe
the Pore-Throat Structure of Tight Sandstones. Pet. Explor. Dev..

[26] Liu K. (2025). A New Method
for Characterizing the Pore-Throat Structure of Tight Sandstone Based
on Nuclear Magnetic Resonance and Constant-Rate Mercury Intrusion. Spec. Oil Gas Reservoirs.

[27] Li S., Chen D. X., Yang Z. Q., Wang Q. C., Wang Y. C., Wang Y. Q., Chen Y. H., Li N., Zhang L., Qu L. B. (2026). Pore-Throat Combination Characteristics
and Their Impact on Fluid
Mobility in Water-Bearing Tight Sandstones. ACS Omega.

[28] Liu X. F., Kang Y. L., Li J. F., Chen Z. J., Ji A. Z., Xu H. W. (2020). Percolation
Characteristics and Fluid Movability Analysis in Tight
Sandstone Oil Reservoirs. ACS Omega.

[29] Zhang X. P., Liu Y. Q., Zhou L., Zhong C. R., Zhang P. F. (2023). Study on
Matrix Damage and Control Methods of Fracturing Fluid on Tight Sandstone
Gas Reservoirs. ACS Omega.

[30] Xin X. Z., Zhao J. M., Xu Y. W., Jin C. H., Luo Z. F., Wang Y. M., Fu H. R. (2024). Study on the Spatial
Damage Characteristics
of Working Fluid in a Sandstone Reservoir and an Unplugged Fluid System. ACS Omega.

[31] Jiang Z. F., Zhu Y. M., Li P., Wang Y., Xiang J. (2023). Applicability
of Nuclear Magnetic Resonance Experiment in Analyzing Pore and Fluid
Distribution Characteristics of Tight Sandstone: A Case Study in the
Julu Area, Bohai Bay Basin, China. ACS Omega.

